# Design and Evaluation in eHealth: Challenges and Implications for an Interdisciplinary Field

**DOI:** 10.2196/jmir.9.2.e15

**Published:** 2007-05-27

**Authors:** Claudia Pagliari

**Affiliations:** ^1^Division of Community Health Sciences (General Practice Section), University of EdinburghEdinburghUnited Kingdom

**Keywords:** Medical informatics, software development, evaluation, interdisciplinarity

## Abstract

Much has been written about insufficient user involvement in the design of eHealth applications, the lack of evidence demonstrating impact, and the difficulties these bring for adoption. Part of the problem lies in the differing languages, cultures, motives, and operational constraints of producers and evaluators of eHealth systems and services. This paper reflects on the benefits of and barriers to interdisciplinary collaboration in eHealth, focusing particularly on the relationship between software developers and health services researchers. It argues that the common pattern of silo or parallel working may be ameliorated by developing mutual awareness and respect for each others’ methods, epistemologies, and contextual drivers and by recognizing and harnessing potential synergies. Similarities and differences between models and techniques used in both communities are highlighted in order to illustrate the potential for integrated approaches and the strengths of unique paradigms. By sharing information about our research approaches and seeking to actively collaborate in the process of design and evaluation, the aim of achieving technologies that are truly user-informed, fit for context, high quality, and of demonstrated value is more likely to be realized. This may involve embracing new ways of working jointly that are unfamiliar to the stakeholders involved and that challenge disciplinary conventions. It also has policy implications for agencies commissioning research and development in this area.

## Aims and Origin of This Article

This paper represents a personal viewpoint based on a nonsystematic review of the literature and the experience of observing and participating in the design, evaluation, and analysis of health informatics interventions. It originated as a briefing document for members of a multidisciplinary team of clinicians, researchers, and software designers, which was designed to foster shared understanding and plan a program of formative and evaluative work. The paper draws on existing literature advocating interdisciplinary methods in medical informatics but focuses on generating a dialogue between software developers and researchers working in this area.

The article begins by considering the increasing heterogeneity of the field, the need for multiple research perspectives, and the implications of scientific subcultures; it discusses the importance of research for ensuring that new eHealth technologies are adopted and effective; it highlights common concepts and methods in software design and health services research; and it then considers the benefits, challenges, and facilitators to interdisciplinary collaboration.

For the purposes of this paper, the term *eHealth* is used broadly as a synonym for health informatics or medical informatics and health services research for health technology assessment and health systems research.

## Out of the Basement: Changing Stakeholders in Medical Informatics

Not so long ago, medical informatics was largely the preserve of computing professionals and managers due to its focus on aspects of information technology “hidden” beneath the surface of health care organizations, such as operating systems, architectures, and databases. While epidemiologists were quick to harness the potential of electronic patient records for research and disease surveillance, it was the growth in practice-based computing during the 1990s that increased awareness of information technologies among clinical stakeholders and saw their gradual integration into the processes of care. Among the general public, awareness of eHealth, as the field is becoming known [1], has burgeoned since the turn of the 21st century, paralleling access to the Internet and the proliferation of Web-based health and lifestyle resources. This is reflected at the policy level, where governments have become increasingly interested in the potential of information and communications technologies to improve the organization and delivery of health services and to support patient empowerment for self-care [2]. In the United Kingdom, for example, the National Health Service (NHS) National Programme for Information Technology is gradually bringing the health service into people’s homes via initiatives such as NHSDirectOnline and NHSHealthSpace, which offer not only information but also opportunities for electronic consulting and personal health care organization (eg, records, appointments) [3].

Reflecting this societal trend, academic involvement in medical informatics has become ever more interdisciplinary, with growing participation by the social, economic, and legal sciences (eg, around managing change, ethics, and cost-effectiveness) and the emergence of translational fields such as bioinformatics that promise to challenge existing medical models. At the same time, the boundaries between scientific, policy, and commercial areas of research and development are becoming grayer, as academia and industry respond to government funding opportunities and the policy community responds to emerging evidence and new technologies.

**Figure 1:**
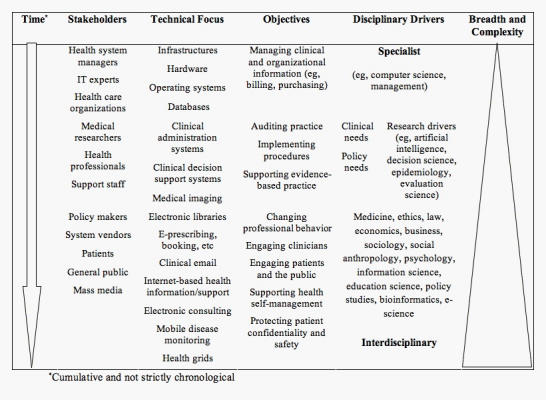
The increasing breadth and complexity of eHealth in terms of the stakeholders, technologies, objectives and disciplines involved

Growing use of the term *health informatics*, in preference to medical informatics, also reflects a shift toward inclusiveness. Figure 1 represents this shifting landscape in terms of the stakeholders, technical focus, disciplinary drivers, and objectives of medical informatics practice and research. It is not intended as a comprehensive chronological account of the field’s evolution, although the domains reflect observations from previous analyses of the literature [1]. A key change has been the increasing breadth and complexity of the field not only in terms of new technologies but also the perspectives that are being brought to bear in planning, understanding, and evaluating these technologies.

## Scientific Subcultures and Their Implications for Interdisciplinary Working

The increasing heterogeneity of the eHealth field raises challenges for interdisciplinary working and the translation of research to policy and practice. These challenges have to do with the management of nonshared concepts and languages and the values ascribed to different forms of scientific and technological endeavor, within what may be termed the knowledge economy of eHealth.

Despite popular stereotypes about “the” scientific paradigm, different disciplines have evolved uniquely over time and have their own theoretical or applied stance, criteria for appraising quality, and their own ways of working. Although we agree on basic principles of objectivity and methodological adherence, the way in which we see the world and approach new research problems is affected by a host of contextual and historical factors that are specific to individual disciplines, making truly interdisciplinary working difficult to achieve [4].

Unpicking all these factors is beyond the scope of a single paper; however, a useful guide is to be found in the comparison of two important areas of activity that are central to modern eHealth—namely, software design and health services research (HSR). The reason for concentrating on these is that managing their relationship is fundamental to ensuring that eHealth innovations achieve their potential to improve the quality, efficiency, and safety of patient care. This paper focuses on the software development process, with particular reference to user-centered design methods. Its origins lie in the author’s growing appreciation of the nature, value, and limitations of evaluation methods used in the software engineering community and the lack of awareness of these among health services researchers evaluating eHealth resources. While several high-profile documents have explored potential synergies between HSR and the broad field of medical informatics, and these have undoubtedly contributed to quality improvements in some areas, their potential impact across the wider eHealth landscape is far from being realized [5-12]. In practice, many eHealth software developments, and the HSR projects associated with them, take place in the context of short contractual episodes, where neither developers nor health services researchers have the time or incentive to engage in cross-disciplinary learning. As a result, developers and researchers of eHealth regularly work in parallel universes, each regarding the other’s domain of activity as separate and neglecting the potential for useful interaction.

## The Need for More Research in Development

Although developing technical solutions remains central to medical informatics, recent years have seen a growing emphasis on identifying and resolving barriers to implementation. Particular attention has been devoted to understanding so-called people and organizational factors, such as stakeholder resistance to change and the appropriate integration of new technologies into work patterns [13,14]. Two key themes have emerged from this discourse, which have direct relevance for the potential effectiveness of eHealth innovations:

1. The clinical appropriateness and usability of eHealth technologies have been compromised by insufficient end-user engagement in the design process.
2. The effectiveness of emerging eHealth technologies in improving the processes or outcomes of health care is unproven.

To consider the first theme, while there is general consensus among software designers on the importance of engaging users in software design and testing, commercial drivers and a historical focus on product development have meant that this has often been inadequate in the past, resulting in top-down developments whose problems may only emerge after rollout. The health care sector has been particularly prone to such problems in recent years, and there are numerous examples of potentially useful systems that have failed or been abandoned due to unanticipated technical, human, or organizational issues [15-17]. Design flaws can affect the ease of use and reliability of systems and may even be dangerous, creating ill-feeling and reducing clinicians’ willingness to use emerging systems, software, and hardware in practice [18,19]. Even seemingly minor problems with usability or conceptual fit can destabilize the implementation of otherwise highly engineered and valid technologies. The discussion that follows illustrates how developers are rising to this challenge.

To consider the second theme of eHealth technologies being unproven, while research in this area is burgeoning, it remains a fact that there is little reliable evidence to demonstrate the measurable impact, risks, or cost-effectiveness of eHealth innovations, except in a modest number of application areas [20-22]. This creates uncertainty and hence a reluctance on the part of clinicians and policy makers to implement such technologies. Where rigorous research designs have been employed, this has often been in the context of academic studies in which the future sustainability or generalizability of the products being evaluated cannot be assured. Indeed, a recent systematic review of health information technologies demonstrated that of 257 published evaluations, a staggering one quarter emanated from four academic institutions that implemented internally developed systems, while only nine reported on commercially developed systems [22]. 

Tackling these problems requires the application of joint thinking between practitioners in the two fields so as to ensure high-quality, user-informed products of demonstrated effectiveness. However, cultural divides between the traditional software developer and health researcher communities have inhibited this process.

## An Evolutionary Snapshot

Software development represents an application of computer science, a field rooted in engineering and mathematics. Although it has drawn on philosophy (eg, semantics, logic) and social science (eg, human-computer interface research, social technology studies), its historical focus has been on building machines and the software they require, albeit with ever more complex digital innovations such as the Internet and intelligent agents. This focus on product development has led to a close alliance with the business and service sectors, and, although basic science is highly valued, there has been an understandable emphasis on applied research and development within university curricula. Within the workforce itself, economic drivers prioritize the production of resources that meet key functionality criteria and client-defined requirements within commercially viable time frames. Evaluation often takes a lower priority, and rapid application development using small convenience samples of users is common [23]. 

HSR is an interdisciplinary field concerned with the scientific study of the structure, processes, and effects of health services, technologies, and policies. This harnesses traditionally medical research approaches from epidemiology and clinical science, alongside the social and economic sciences, utilizing a mixture of quantitative and qualitative methodologies appropriate for the specific problem under investigation. It is closely allied to the evidence-based medicine movement, which holds that clinical practice should be driven by evidence of what interventions work best and for whom. As well as measuring impacts, such research is also about enquiry. For example, it may explore the needs of particular stakeholder groups or demographic patterns of health and health care utilization in order to identify the place of a potential new intervention or examine the reasons why an intervention is more easily adopted or more effective in different contexts. A defining characteristic of this field is the strong emphasis on methodological rigor. From randomized controlled trials to qualitative case studies, the focus is on detailed planning and recording of procedures and on transparent, theoretically informed participant sampling and data analysis. This area is less influenced by commercial drivers, although there is a strong emphasis on research that addresses health service policy needs.

Thus, software design is mainly concerned with developing interventions, and HSR, with evaluating them. But look closer and the reality is not so clear cut. In fact, much of HSR is geared toward informing the design of new interventions, including eHealth technologies, while rigorous software design encompasses evaluation processes that would be very familiar to health services researchers.

However, within these two communities there has been a mutual lack of awareness of each others’ theoretical stance, motives, and modus operandi, exacerbated by differences in language, epistemologies, and the representation of concepts. This reflects the origins of the two disciplines and the funding environment, which place different expectations on eHealth design and research projects.

## Compatibilities in Models and Methods of Software Development and Health Services Research

While various academic approaches have been applied to the study of software design and diffusion (eg, in the management literature), in the context of this paper the compatibilities between process models of software development and HSR are particularly relevant. These compatibilities illustrate the importance of exploration and evaluation for informing developments and quality improvements in both domains, the value of user engagement in this process, and the natural progression to assessment of the effects.

### Lifecycle Models as an Exemplar from Software Engineering

**Figure 2:**
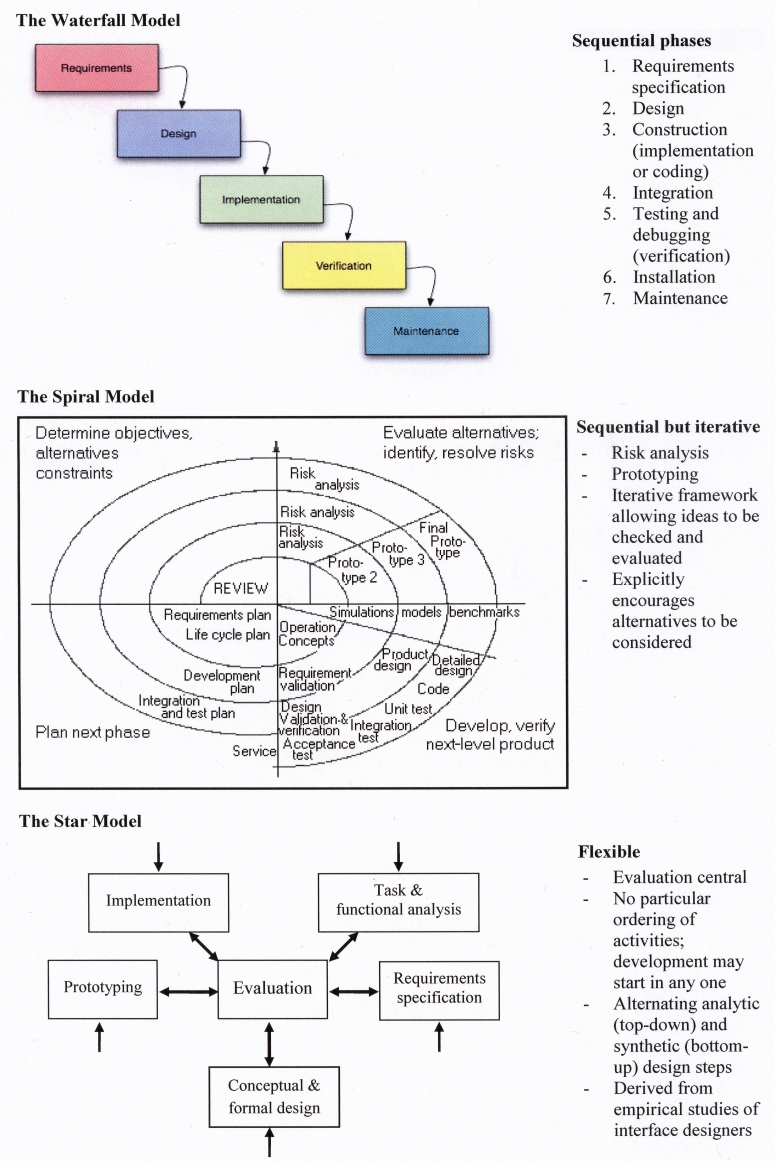
Key software lifecycle models: Waterfall [25], Spiral [26], Star [27] model

**Figure 3:**
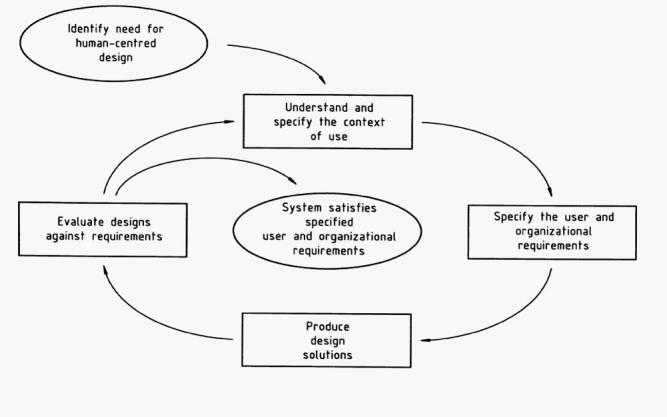
ISO 13407 standard for human-centered design processes for interactive systems

Within software engineering, numerous models have been proposed to describe the process by which products should be designed and tested to ensure they are fit for purpose in the intended setting [24]. Particular parallels with HSR can be found in a category known as lifecycle models, the most common of which are the Waterfall, Spiral, and Star models, referring to the sequence and pattern of substages involved (Figure 2) [25-27]. Of these, the Spiral and Star models are frequently advocated due to their ability to cope with iteration and complexity, although in practice the more sequential Waterfall method is often used [28]. All of these illustrate the codependence of development and evaluation, while the Spiral and Star models emphasize iterative design. Although they have been slow to evolve, software design and development methodologies now almost universally include user-developer interaction for requirements determination, testing, and acceptance activities; indeed, this is a central feature of the Star Model. Figure 3 illustrates the model of user-centered design of the International Organization for Standardization (ISO) that is increasingly adopted when developing interactive systems [29]. The critical feature of this, and other approaches to user-centered design (or usability engineering), is the emphasis on determining users’ needs of the system, understanding the context in which the system will be delivered, and designing products from the ground-up rather than based on developers’ preconceptions or rigid procurement briefs. Such methods are being increasingly advocated, and their successful use is being reported in the medical research literature [30,31]. In some development settings, the user has taken a further step toward the center of the design process; for example, a paradigm employed in the defense sector uses software to directly involve users in developing their own problem-solving intelligent agents [32]. 

### Phased and Iterative Models of Health Services Research

There is little awareness of software lifecycle models among typical health services researchers, yet these are highly compatible with phased approaches to drug development and the evaluation of complex interventions in health care, which emphasize the need for exploratory, explanatory, and pragmatic phases, as illustrated in Figures 4a and 4b [33]. Particular parallels can be seen in the stages of concept formation, needs assessment, and evaluation in the intended setting.

**Figure 4a:**
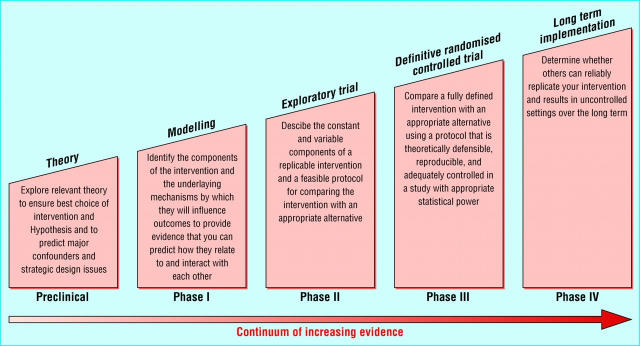
Sequential stages in evaluation of complex interventions (after [33]). Similar steps are used in the evaluation of new drugs, from initial preclinical research through to postmarketing surveillance.

**Figure 4b:**
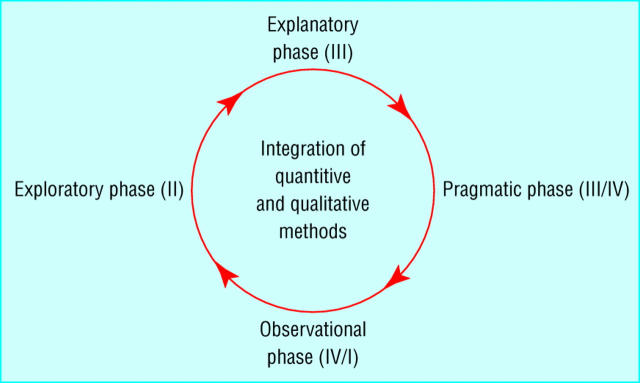
Iterative view of complex intervention evaluation (after [33]). This recognizes that results from individual phases may prompt revisions and repetition.

**Figure 5:**
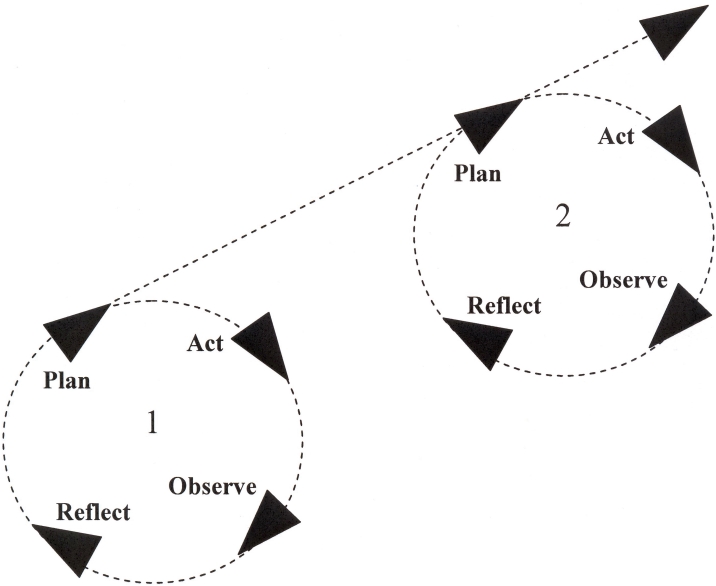
The Action Research Spiral (after [34])

**Figure 6:**
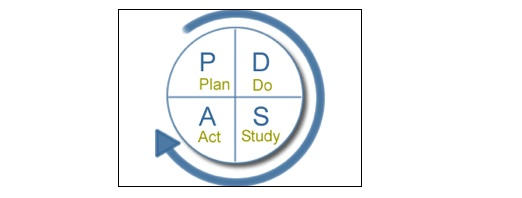
The “Plan-Do-Study-Act” process improvement cycle of total quality management (after [35])

Similarly, models of user-centered design bear a close resemblance to iterative HSR models such as Action Research [34] and Continuous Quality Improvement [35], examples of which are given in Figures 5 and 6. These also conceive of a cycle or series of cycles through which users’ needs are assessed, interventions developed, problems identified, and changes made to the intervention or the management of its delivery. Indeed, these models are advocated within both the health care and software development arenas [36,37]. 

### Overlapping Research Techniques in Health Services Research and Software Design

As well as the parallels between overarching process models, there is considerable overlap in the precise research techniques used in both software development and HSR. While the terminology varies, user-centered design methods such as requirements gathering, observation of task walk-throughs, think aloud protocols, and group-based feedback are similar to HSR methods such as needs assessment, participant observation, semistructured interviews, and focus groups; indeed, these terms are regularly used to describe activities in information technology labs, although the way in which they are applied may be somewhat different. Examples of techniques used in both fields are provided in Table 1 to illustrate areas of overlap and divergence. Differences in the portfolios of methods reflect the somewhat dissimilar (although overlapping) goals of evaluation within software engineering and HSR: the former focusing on optimizing product design and fitness for purpose, and the latter on exploring new phenomena, generating hypotheses, demonstrating impact, or informing policy. Most noteworthy is the absence of rigorous impact assessment (controlled trials) within the scope of software engineering, in contrast to its high status within HSR. An important differentiating feature not reflected in Table 1 is the heavy emphasis on theoretical sampling and meticulous time-intensive approaches to qualitative data analysis within HSR. This contrasts with the more rapid identification of needs and responses common in development projects and the often unstructured and iterative nature of the design process. Nevertheless, software engineering can also involve quantitative usability techniques that draw on cognitive psychology. When these are employed, it is often in a highly systematic manner, involving multiple measurements and theoretically based analysis, although the objectives are best met with depth studies of small numbers of users. These methods have great value for the understanding of human errors and information processing, and, although there is little knowledge of them within the HSR community, they are increasingly being reported in medically indexed journals [38-40].

**Table 1:** Examples of methods in user-centered design and health services research

**Software and Usability Engineering**	**Health Services Research**
**Needs Assessment** (conceptual, formative) Requirements gathering: assessment of prototypes/simulations; user interviews	**Needs Assessment** (conceptual, formative) Interviews; document analysis; telephone or postal surveys; focus groups; observation; discrete choice simulations
**Assessment** (primarily formative) Heuristic evaluation; cognitive walk-throughs; formal usability inspection; pluralistic walk-throughs; feature inspection; consistency inspection; standards inspection; guideline checklists; thinking aloud protocol; prototyping; co-discovery methods; question asking protocol; performance measurement; gaze tracking; ethnographic study / field observations; surveys; questionnaires; journaled sessions; self-reporting logs; remote usage observation; screen snapshots; blind voting; card sorting; archetypal research, action research	**Assessment** (formative or summative) Experimental and quasi-experimental designs (eg, randomized controlled trial; controlled before and after study; interrupted time series; case control study; cost-benefit analysis) Qualitative outcomes assessment: rigorous qualitative data analysis using sociological methods (eg, ethnographic studies) Observation/exploration: remote (eg, epidemiological, records-based); direct (eg, participant or nonparticipant) Participative evaluation (eg, action research / continuous quality improvement)

Note: This is a non-exhaustive list drawing on several taxonomies that is designed merely to illustrate some of the common and distinctive techniques used in both disciplines.

### Integrated Models in Medical Informatics

**Figure 7:**
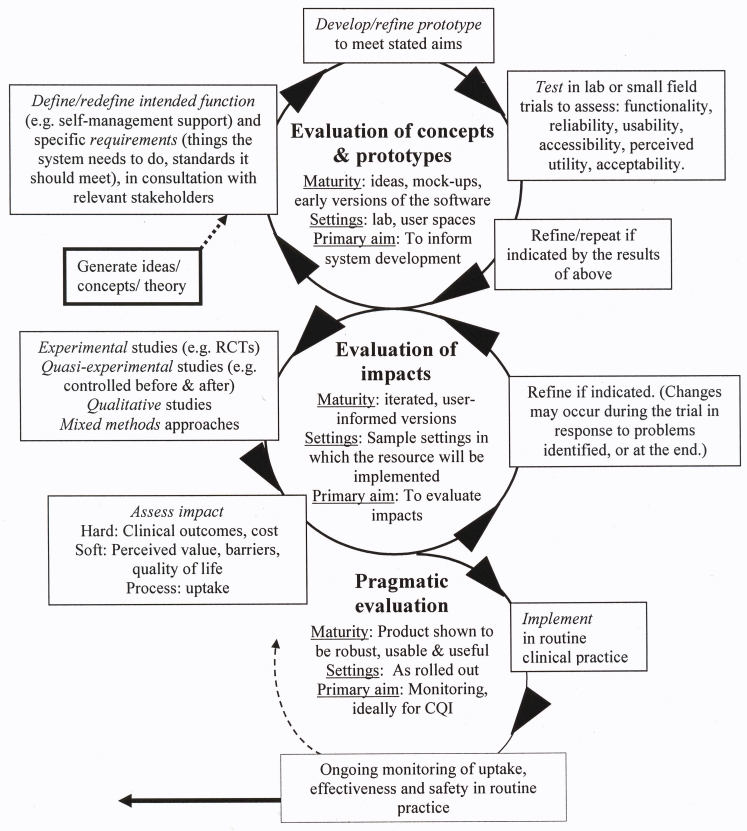
An idealized framework for evaluating emergent eHealth resources at different stages of development and implementation

Within the interdisciplinary field of medical informatics, hybrid models have appeared that draw on both traditions, an example of which is offered in Figure 7. Importantly, there is a growing acceptance that evaluation should ideally be approached as a longitudinal process occurring through a series of overlapping and iterative stages relevant to the maturity of the technology in its lifecycle, from initial conception to rollout. Various authors have attempted to represent these stages and to provide taxonomies of research methods appropriate to each [41]; however, three broad phases of activity may be discerned. The first of these involves drafting new interventions based on an assessment of stakeholder needs and theory, and testing these with content experts and users to ensure they fulfil these needs and are technically robust (concept and prototype evaluation). The second involves assessing the impact of the innovations on the processes and outcomes of care in selected target settings, including hard measures such as efficiency, clinical status, cost, and error rates, softer measures of attitudes and satisfaction, and qualitative outcomes (outcomes evaluation). A third phase involves evaluating systems after rollout (pragmatic evaluation), for example, to assess variations in uptake, reported errors, technical problems, stakeholder satisfaction, or longer term impacts on process or outcome indicators. At each of these stages, the model should allow for the results of the research to inform continuous quality improvements. In practice, key stages are often neglected, reducing both the quality and adoption of new eHealth products.

## Benefits, Challenges, and Facilitators of Interdisciplinary Collaboration

### Potential Benefits

The types of conceptual and methodological commonalities outlined above demonstrate that collaborative working between eHealth services researchers and software developers is both possible and appropriate. In addition to producing better and safer interventions [18,19,42], effective collaboration will strengthen the quality of evaluations and enhance the evidence base in this area, thus facilitating policy and purchasing decisions. While involvement in formal research may seem like a hindrance to developers, particularly in the commercial environment, economic as well as intellectual benefits may accrue by demonstrating that systems are effective, cost-effective, and safe, as well as technically robust, accessible, and usable [8]. Indeed, the value of so-called evidence-based business is being increasingly recognized in the technology sector [43]. At the same time, rigorous qualitative studies can demonstrate the acceptability and utility of new tools to users as well as features of the setting or implementation methods that may influence their adoption. For health services researchers, the ability to enter the world of the developer presents valuable opportunities to influence the scoping, design, and evaluation processes used to develop electronic health care interventions that may then be subjected to clinical trials, thus ensuring conceptual fit and minimizing the risk of confounding by suboptimal functionality or usability [44]. It also offers a new skill set that may help researchers to recognize cognitive barriers to technology adoption and thus aid interpretation of descriptive or evaluative data. An added benefit for both groups is the increased ability to publish that comes from adopting systematic and replicable sampling and analytic methods in the course of user-centered design, thus facilitating dissemination to both audiences [45].

### Challenges

There is a need to move the current agenda away from a state of parallel working, which is common in multidisciplinary projects involving the computing and medical sciences, to one of truly interdisciplinary working. This requires an appreciation of each others’ terminologies, goals, and methods and the sharing of experiential learning about the benefits and limitations of alternative approaches. It also calls for the generation of a new breed of transdisciplinary experts familiar with the implementation of both skill sets and able to combine them in novel ways in order to achieve maximum value for eHealth research and development. Overcoming cultural and methodological divisions between disciplines represents a major challenge. There is a natural inclination to remain pure to concepts and methods that may have taken many generations to evolve, and questions arise around how far to take joint approaches before compromising each discipline’s ability to demonstrate their specific expertise [4]. There is also a fundamental tension between the need to innovate, which may require conceptual leaps of faith and rapid developments, and the pressure to adopt methodologically robust standards of scoping, sampling, and evaluation that may be time-consuming and of questionable value at the early stages of prototyping. This can create antagonism and defensiveness in both camps, thus inhibiting potential synergies. Successful interdisciplinarity therefore requires the establishment of trust and mutual respect in addition to methodological pluralism, and this represents a challenge, particularly where the opportunity to become embedded in the other’s world is not available. Importantly, different approaches will be appropriate for addressing different objectives in different settings and at different stages of software maturity, and a challenge for project leaders and commissioners is to develop a deep enough understanding of multiple methods to be able to tailor these appropriately. For example, controlled trials may be ideal for studying the impact of eHealth systems on measures of clinical outcome or efficiency, but they are poorly suited to exploring social, contextual, or technical barriers to adoption and certainly will have little to offer developers designing a new Web interface. Conversely, think aloud methods may be extremely useful for assessing the usability of a decision support tool but say very little about its clinical validity or effectiveness [46-48]. The value attributable to different forms of evidence thus varies depending on the context in which it is used, although adherence to high standards of data collection, analysis, and reporting is a universal objective. It should also be recognized that academic incentives favoring controlled studies (eg, research funding, impact ratings) may create a conflict for health services researchers wishing to engage in applied and collaborative projects.

### Facilitators

The move toward more holistic training in medical informatics advocated by bodies such as the American Medical Informatics Association represents one step to achieving these goals [5,9], and there is evidence of a trend toward increasing pluralism in the objects of evaluation projects, which may signal a move toward greater interdisciplinarity [48]. Pockets of transdisciplinary working are emerging as eHealth becomes a target of research, for example, within academic units of human-computer interaction and science and technology studies, while the field of information science has a long tradition of research exploring socioeconomic and organizational influences on technology development and adoption, from which eHealth researchers and developers have much to learn [49]. Nevertheless, few individuals working on eHealth projects have received formal cross-disciplinary training and many are doing so as part of a broader portfolio of projects (often on short-term contracts), restricting their motivation to invest in learning the methods and modus operandi of their disciplinary counterparts.

There is a need to influence potential funders, who have traditionally held different expectations for design and evaluation projects in terms of expected outputs (eg, new products vs new knowledge) and methodologies (eg, user-centered design vs studies of clinical impact) and who may underestimate the value of unfamiliar approaches. Importantly, it is essential for those commissioning new eHealth products to be aware of the value of high-quality evaluation during the development process and to allow the time and resources for this to be built into the project. While research agencies are coming to recognize the need to pay attention to usability engineering and other software design methodologies when developing eHealth tools for research, the message of added value needs to be more widely communicated. This is particularly so in view of the revenue currently being invested in health-related websites, many of which are often of poor quality and unknown effectiveness [50], and the vast expenditure being devoted to eHealth technologies by governments worldwide [51]. Without this understanding, those commissioning products in this area will continue to be unwittingly complicit in the process of suboptimal design, while those commissioning evaluation will risk poor value for money if the questions asked are inappropriate or the research methods not suited to answering them [52-54].

## Conclusions

Designing effective eHealth systems and services requires the application of expertise from diverse fields and will benefit from interdisciplinary collaboration. This may be eased by increasing familiarity with each others’ terminologies, theoretical bases, and research methods, with the ultimate objective of achieving transdisciplinary working. There is sufficient overlap in the techniques and concepts employed within the software design and HSR communities to make this a reality, but realizing this requires the development of mutual trust and respect for each others’ aims, epistemologies, and contextual drivers, as well as a willingness to step outside traditional working boundaries. New funding strategies that recognize the value of alternative methodologies and of joint working between developers and evaluators are also called for.

This paper has merely scratched the surface of a wider debate on the value of interdisciplinarity for improving the quality and effectiveness of eHealth, although it is hoped that by highlighting the potential synergies between HSR and software development it will help to provoke constructive dialogue between these two communities. Maximizing the potential of eHealth also requires the involvement of a wider constituency of disciplinary experts, including social, management, and legal scientists, all of whom have a stake in the field. Interdisciplinary networks, such as the one managed by the author, offer one means of addressing this need.

## Multimedia Appendix

PowerPoint slides of MedNet 2006 presentation 
						

## Abbreviations

HSR: health services research
