# Supplementary material for: Design and Evaluation in eHealth: Challenges and Implications for an Interdisciplinary Field
Source: J Med Internet Res. 2007 May 27;9(2):e15. doi: 10.2196/jmir.9.2.e15 (PMC1913937; doi:10.2196/jmir.9.2.e15)
Supplement: Supplementary file 1 [file jmir_v9i2e15_app1.ppt]

## Slide 1
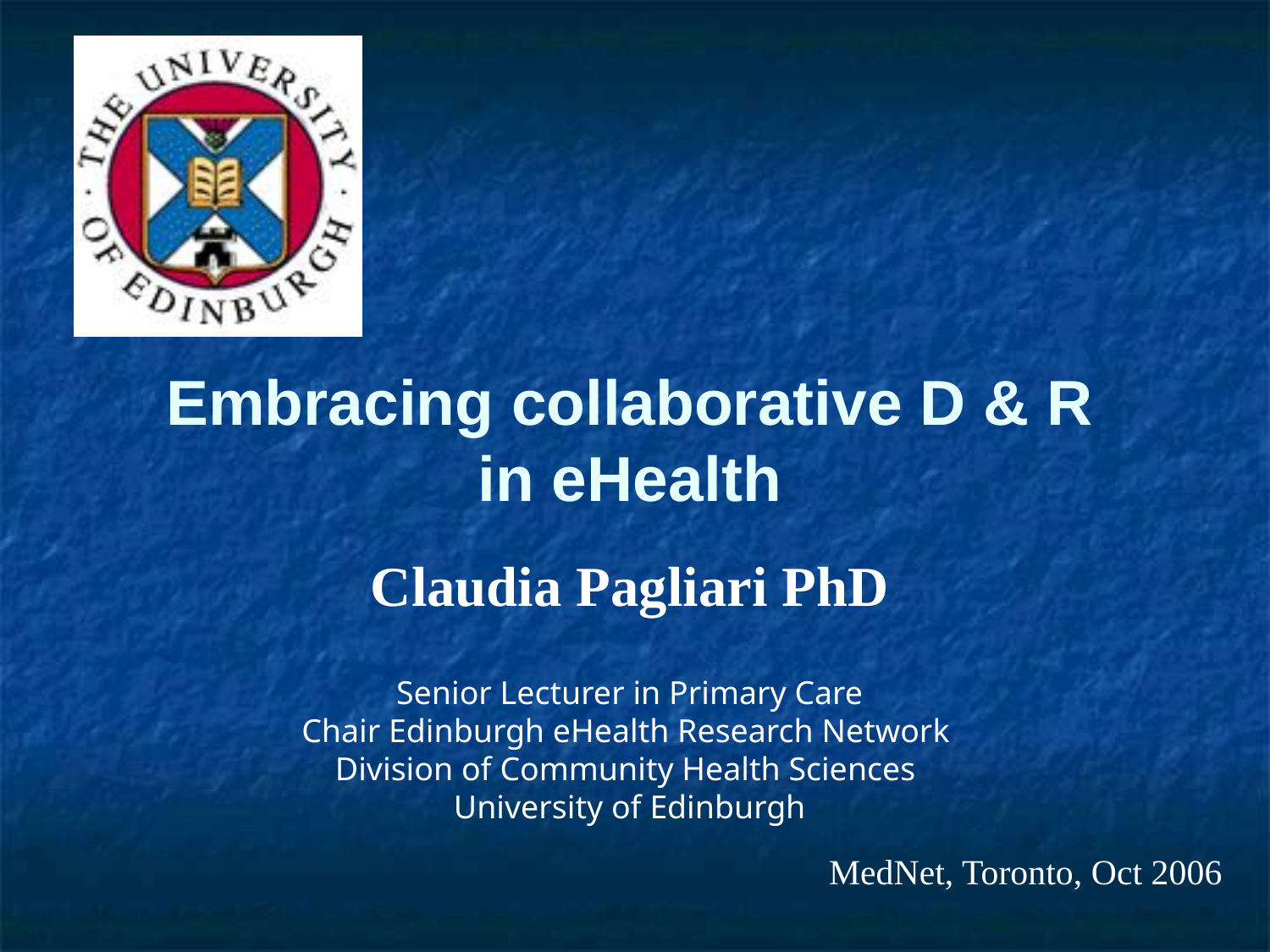

Embracing collaborative D & R in eHealth
Claudia Pagliari PhD
Senior Lecturer in Primary Care
Chair Edinburgh eHealth Research Network
Division of Community Health Sciences
University of Edinburgh
MedNet, Toronto, Oct 2006

## Slide 2
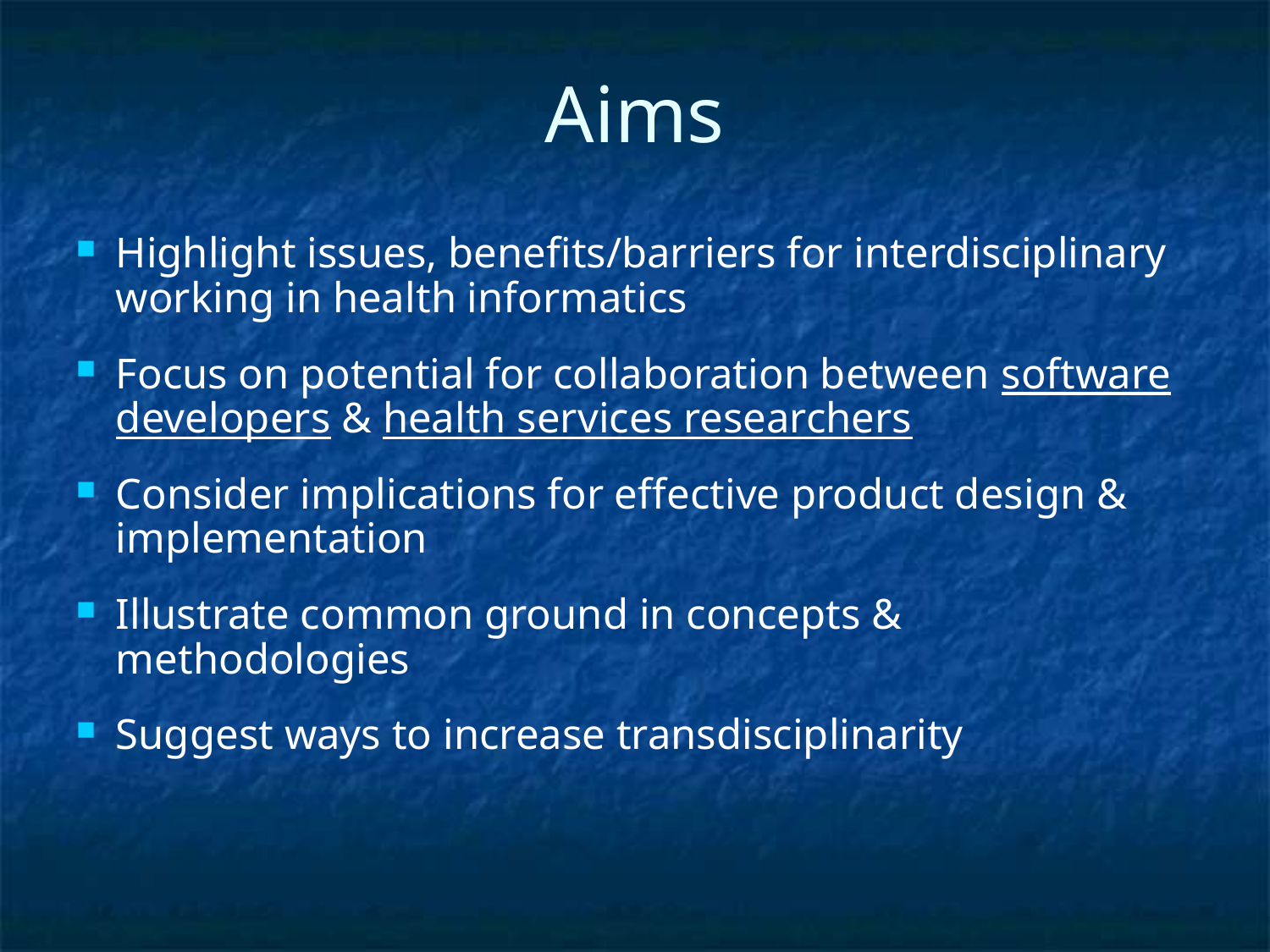

# Aims
Highlight issues, benefits/barriers for interdisciplinary working in health informatics
Focus on potential for collaboration between software developers & health services researchers
Consider implications for effective product design & implementation
Illustrate common ground in concepts & methodologies
Suggest ways to increase transdisciplinarity

## Slide 3
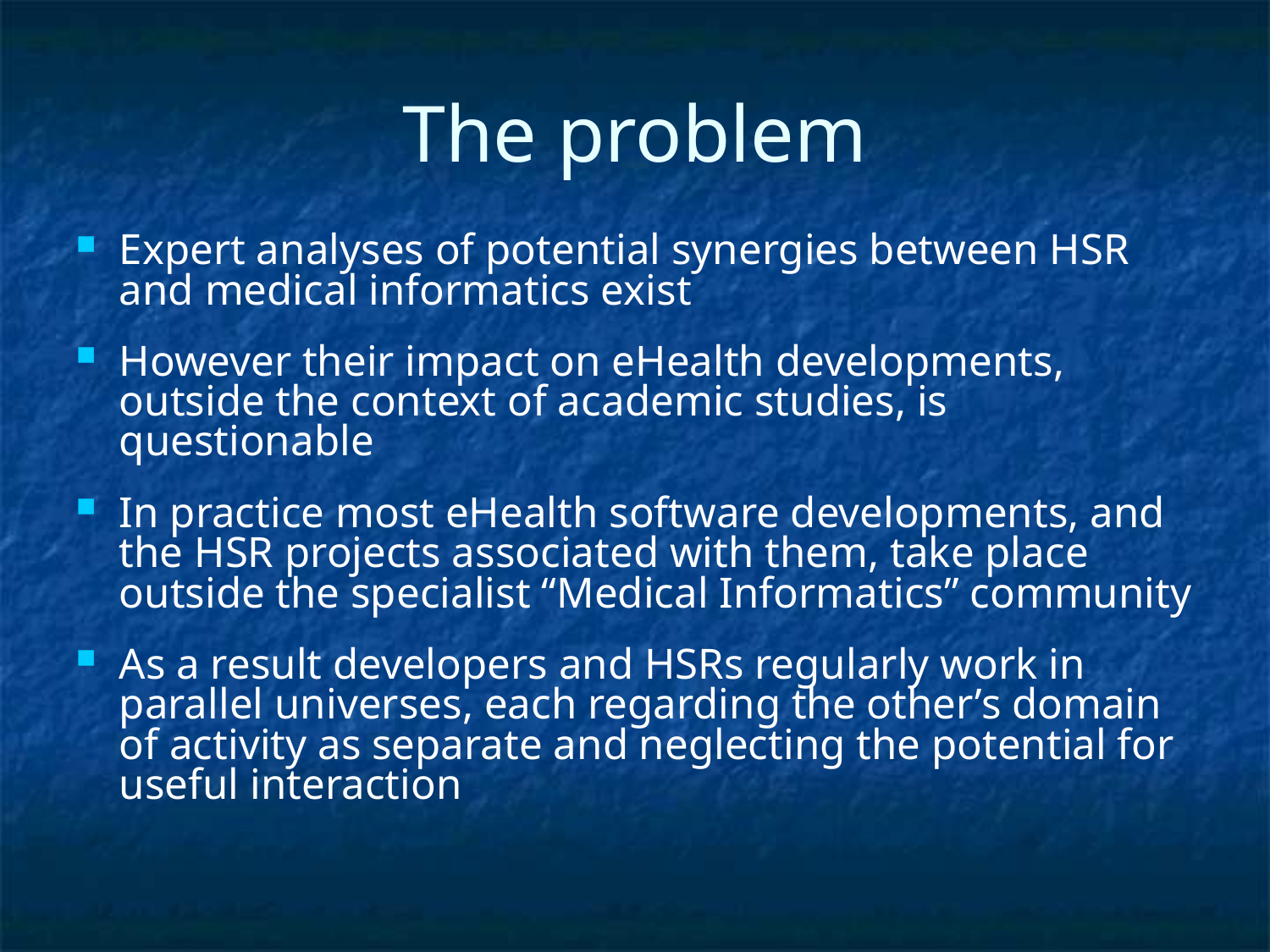

# The problem
Expert analyses of potential synergies between HSR and medical informatics exist
However their impact on eHealth developments, outside the context of academic studies, is questionable
In practice most eHealth software developments, and the HSR projects associated with them, take place outside the specialist “Medical Informatics” community
As a result developers and HSRs regularly work in parallel universes, each regarding the other’s domain of activity as separate and neglecting the potential for useful interaction

## Slide 4
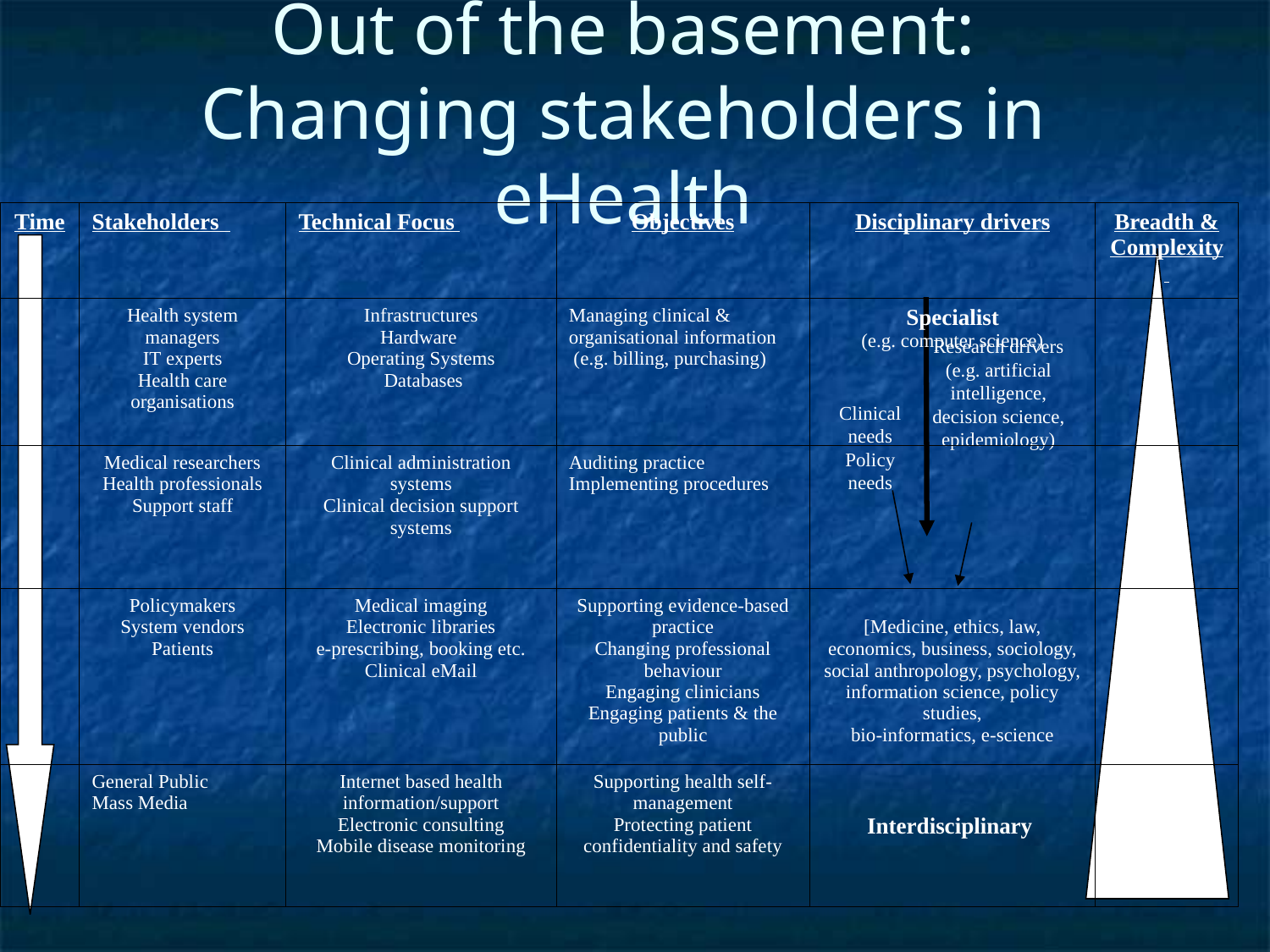

Out of the basement:
Changing stakeholders in eHealth
#
| Time | Stakeholders | Technical Focus | Objectives | Disciplinary drivers | Breadth & Complexity |
| --- | --- | --- | --- | --- | --- |
| | Health system managers IT experts Health care organisations | Infrastructures Hardware Operating Systems Databases | Managing clinical & organisational information (e.g. billing, purchasing) | Specialist (e.g. computer science) | |
| | Medical researchers Health professionals Support staff | Clinical administration systems Clinical decision support systems | Auditing practice Implementing procedures | | |
| | Policymakers System vendors Patients | Medical imaging Electronic libraries e-prescribing, booking etc. Clinical eMail | Supporting evidence-based practice Changing professional behaviour Engaging clinicians Engaging patients & the public | [Medicine, ethics, law, economics, business, sociology, social anthropology, psychology, information science, policy studies, bio-informatics, e-science | |
| | General Public Mass Media | Internet based health information/support Electronic consulting Mobile disease monitoring | Supporting health self-management Protecting patient confidentiality and safety | Interdisciplinary | |
Research drivers (e.g. artificial intelligence, decision science, epidemiology)
Clinical needs
Policy needs

## Slide 5
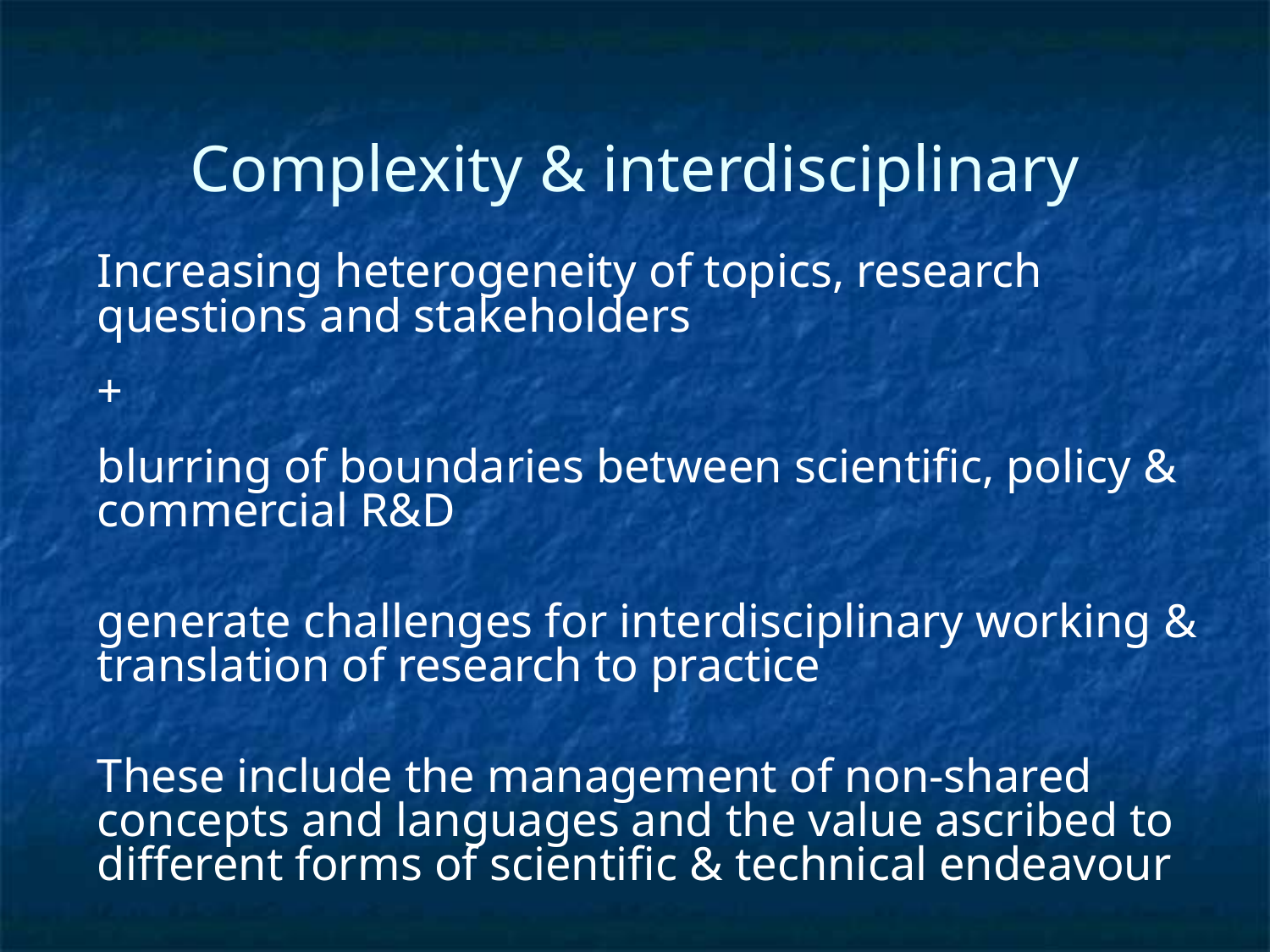

# Complexity & interdisciplinary
Increasing heterogeneity of topics, research questions and stakeholders
+
blurring of boundaries between scientific, policy & commercial R&D
generate challenges for interdisciplinary working & translation of research to practice
These include the management of non-shared concepts and languages and the value ascribed to different forms of scientific & technical endeavour

## Slide 6
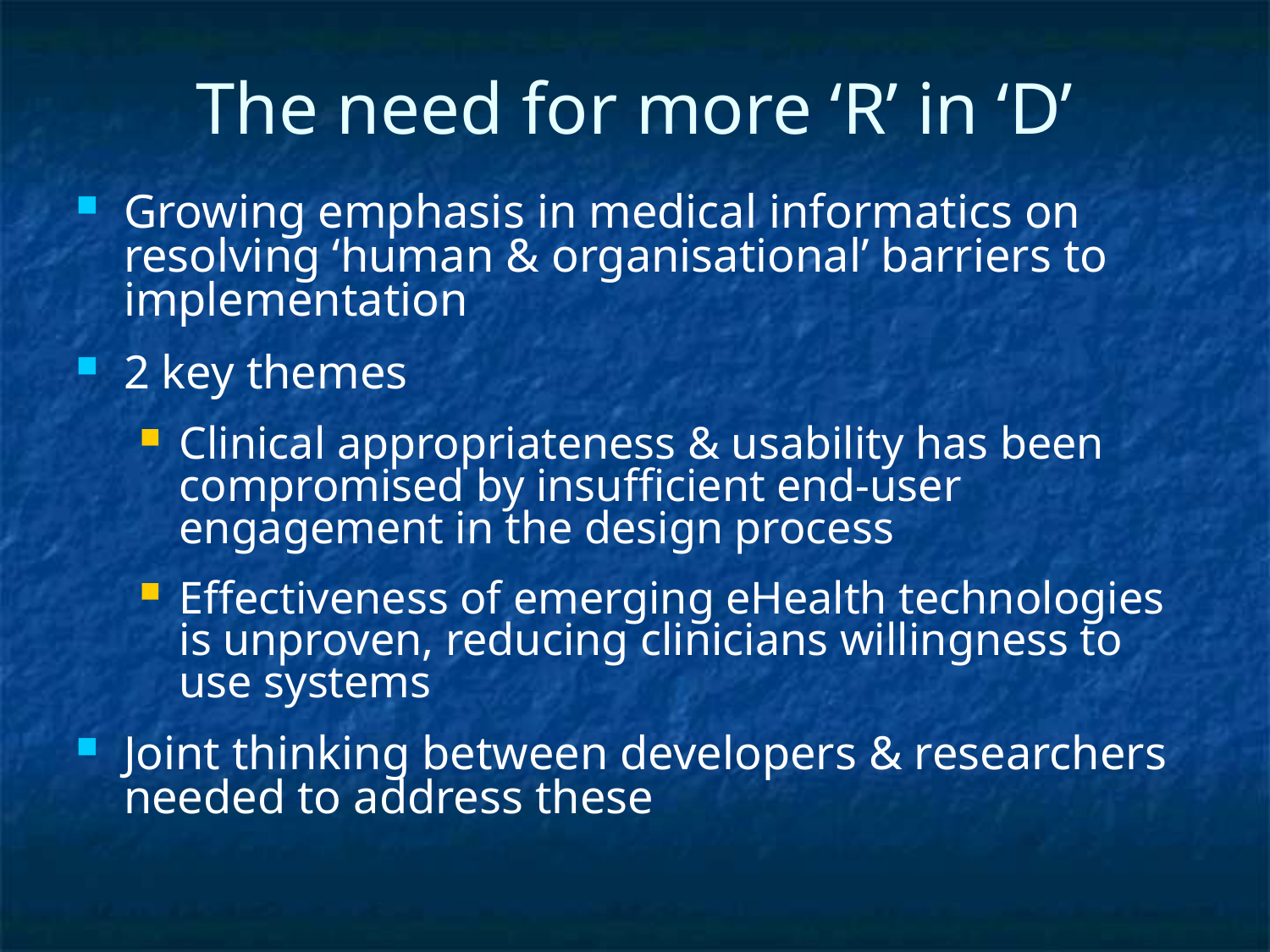

# The need for more ‘R’ in ‘D’
Growing emphasis in medical informatics on resolving ‘human & organisational’ barriers to implementation
2 key themes
Clinical appropriateness & usability has been compromised by insufficient end-user engagement in the design process
Effectiveness of emerging eHealth technologies is unproven, reducing clinicians willingness to use systems
Joint thinking between developers & researchers needed to address these

## Slide 7
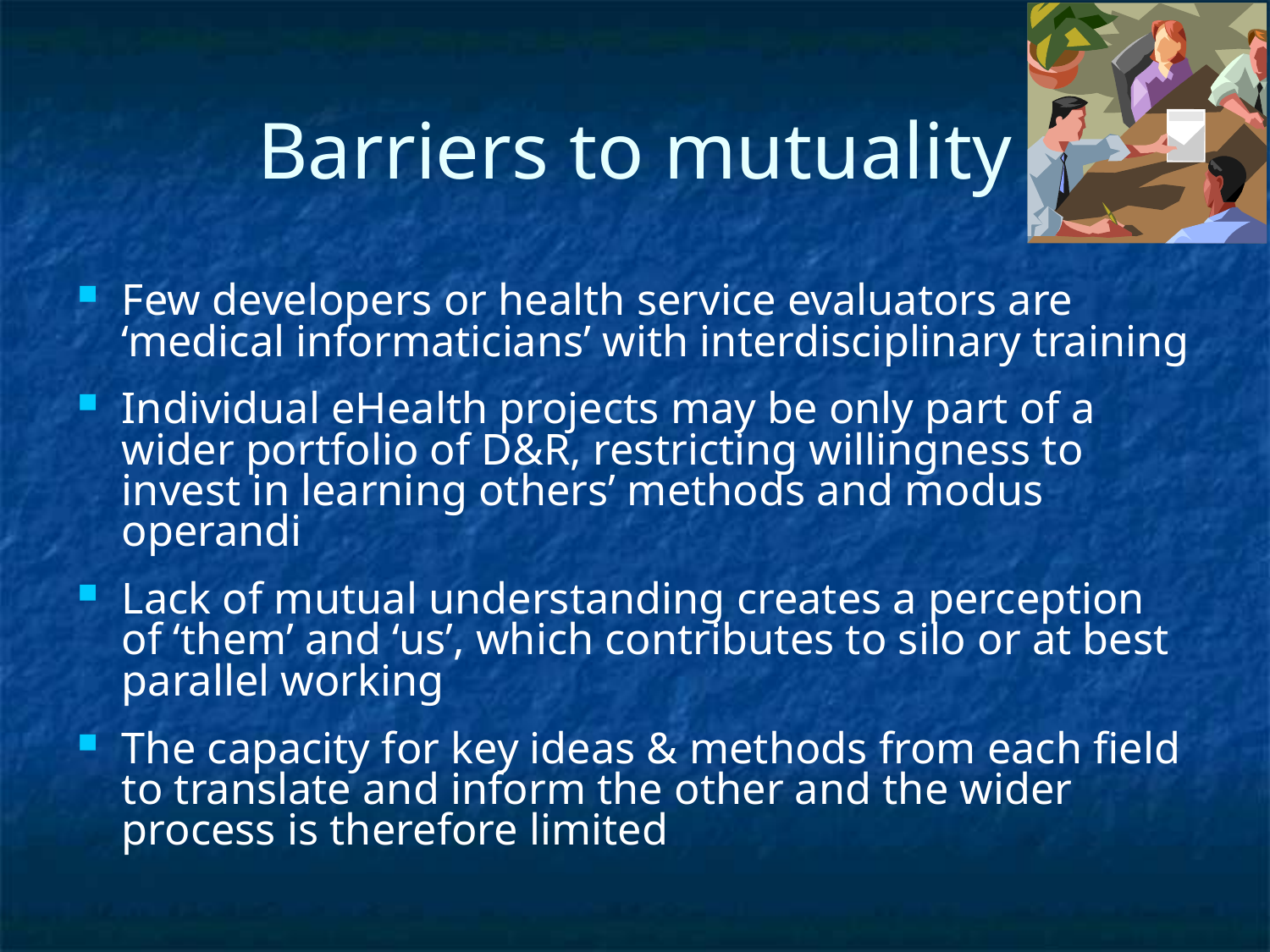

# Barriers to mutuality
Few developers or health service evaluators are ‘medical informaticians’ with interdisciplinary training
Individual eHealth projects may be only part of a wider portfolio of D&R, restricting willingness to invest in learning others’ methods and modus operandi
Lack of mutual understanding creates a perception of ‘them’ and ‘us’, which contributes to silo or at best parallel working
The capacity for key ideas & methods from each field to translate and inform the other and the wider process is therefore limited

## Slide 8
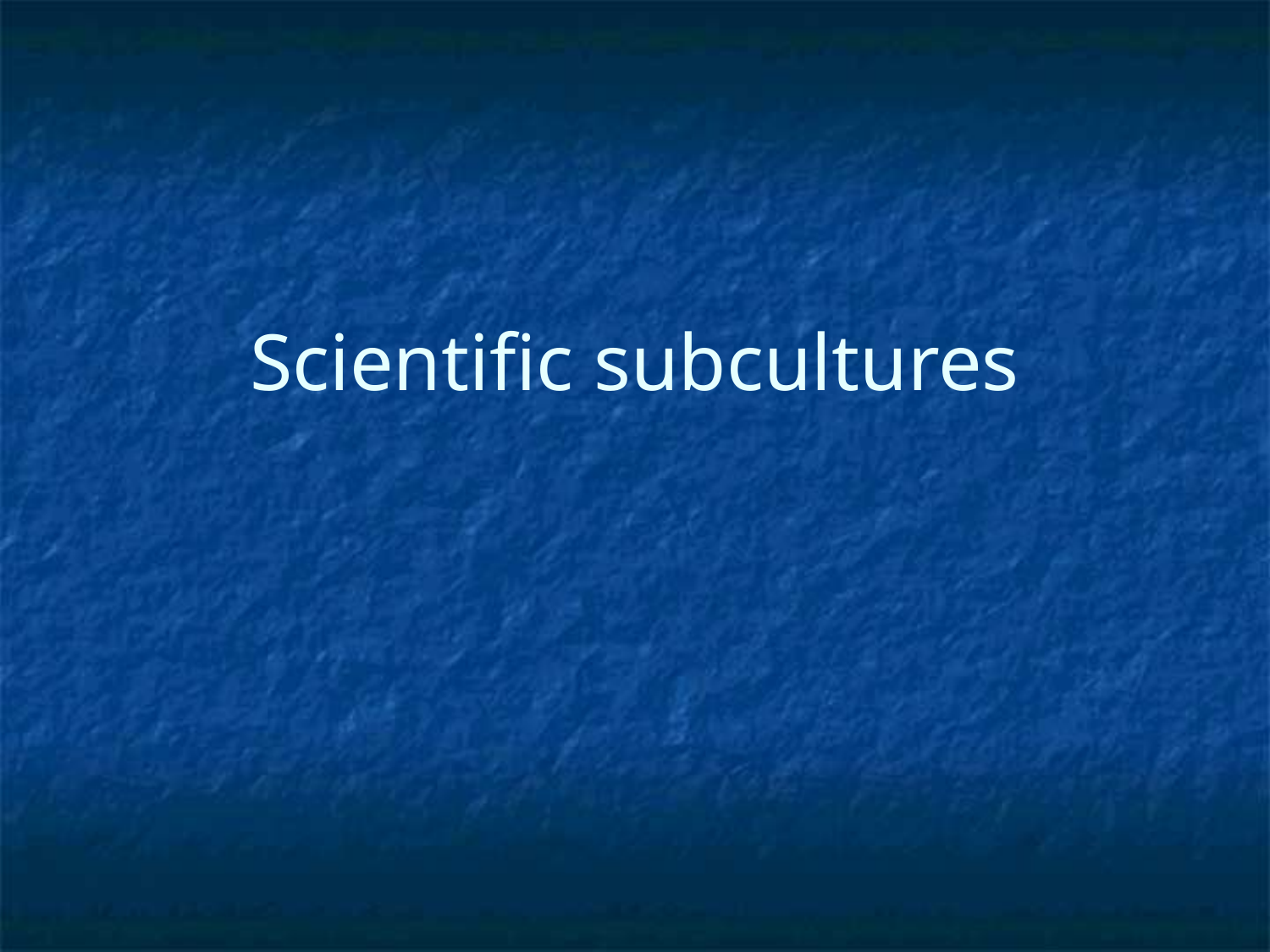

# Scientific subcultures

## Slide 9
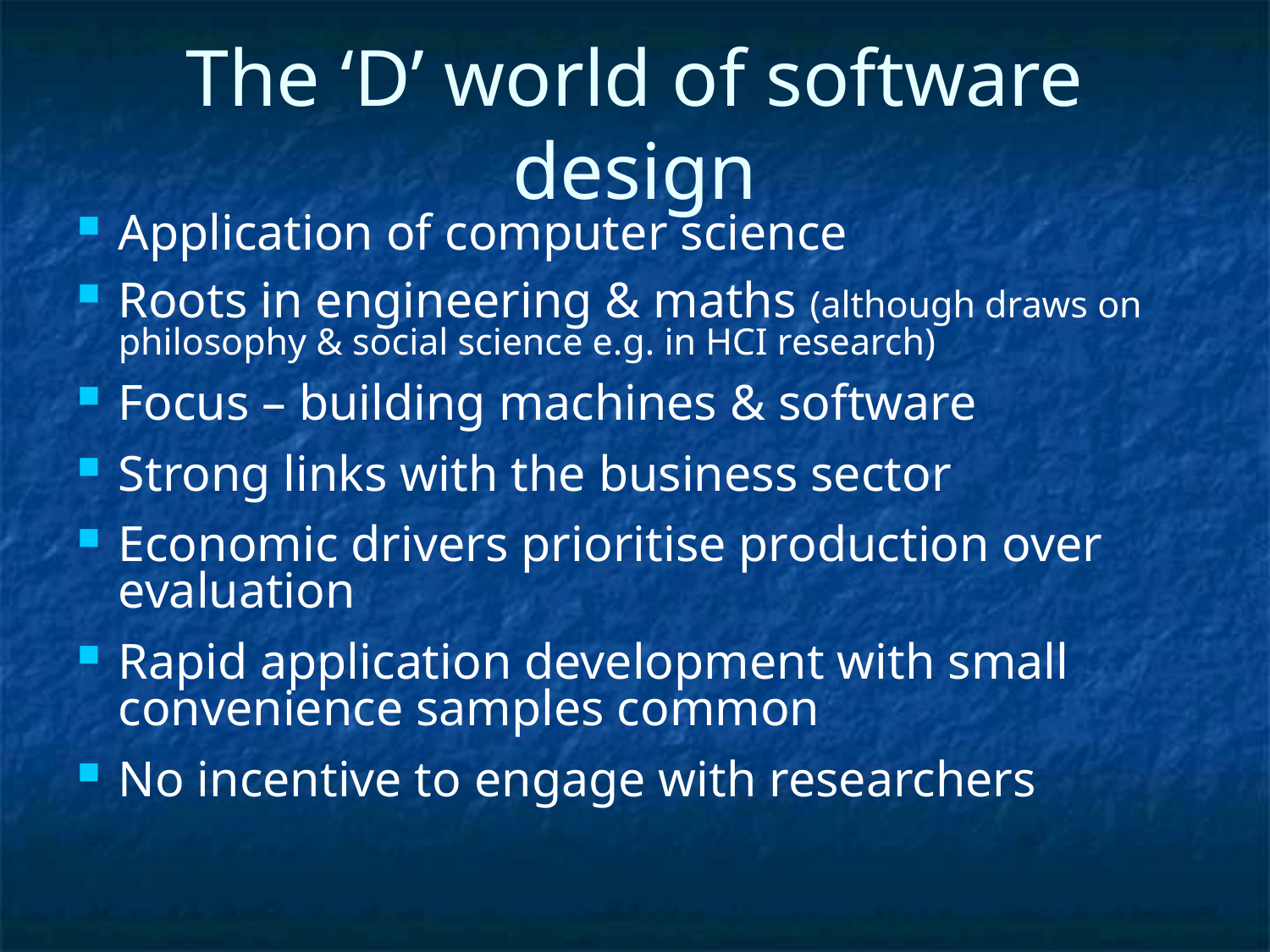

# The ‘D’ world of software design
Application of computer science
Roots in engineering & maths (although draws on philosophy & social science e.g. in HCI research)
Focus – building machines & software
Strong links with the business sector
Economic drivers prioritise production over evaluation
Rapid application development with small convenience samples common
No incentive to engage with researchers

## Slide 10
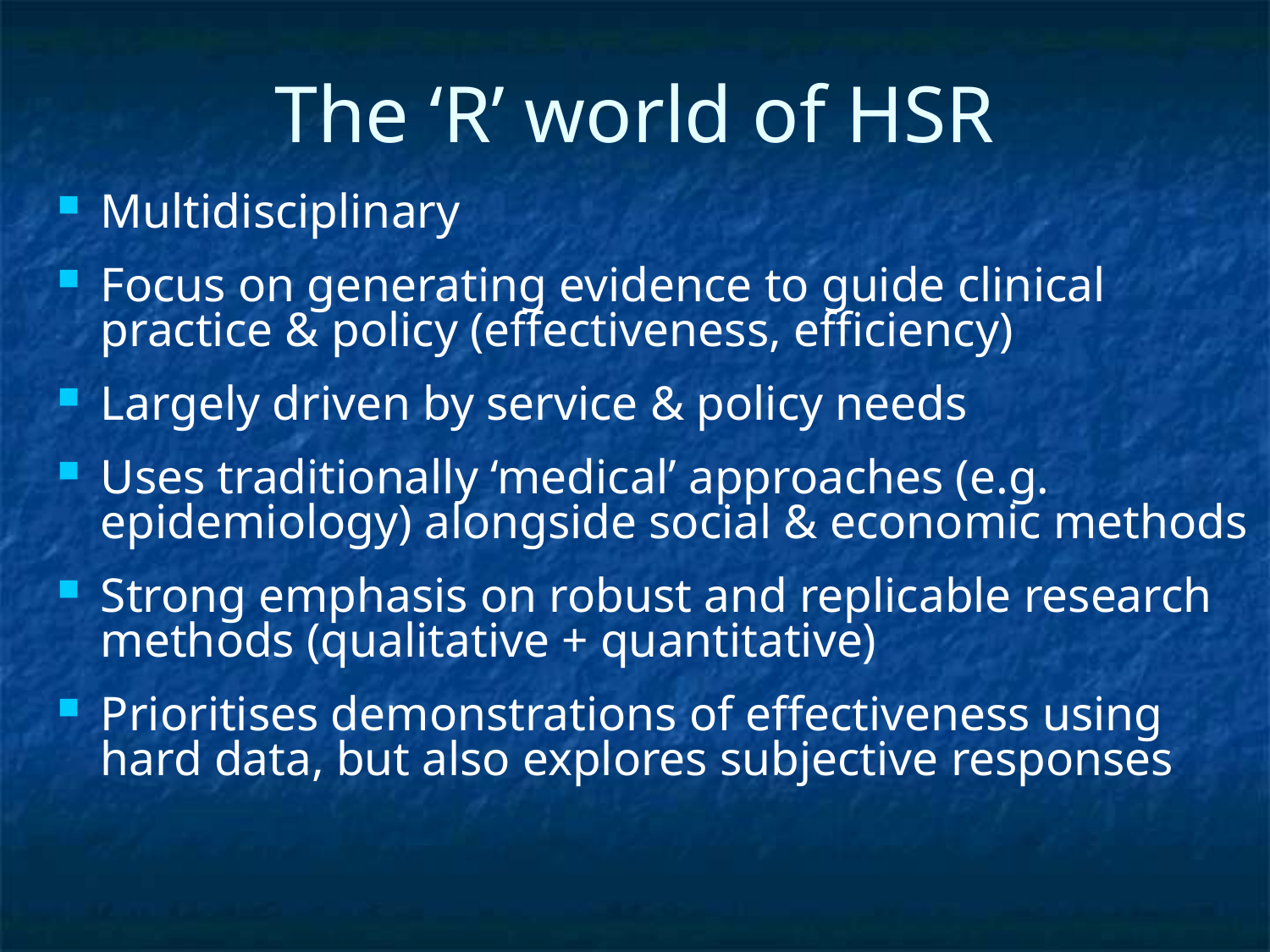

# The ‘R’ world of HSR
Multidisciplinary
Focus on generating evidence to guide clinical practice & policy (effectiveness, efficiency)
Largely driven by service & policy needs
Uses traditionally ‘medical’ approaches (e.g. epidemiology) alongside social & economic methods
Strong emphasis on robust and replicable research methods (qualitative + quantitative)
Prioritises demonstrations of effectiveness using hard data, but also explores subjective responses

## Slide 11
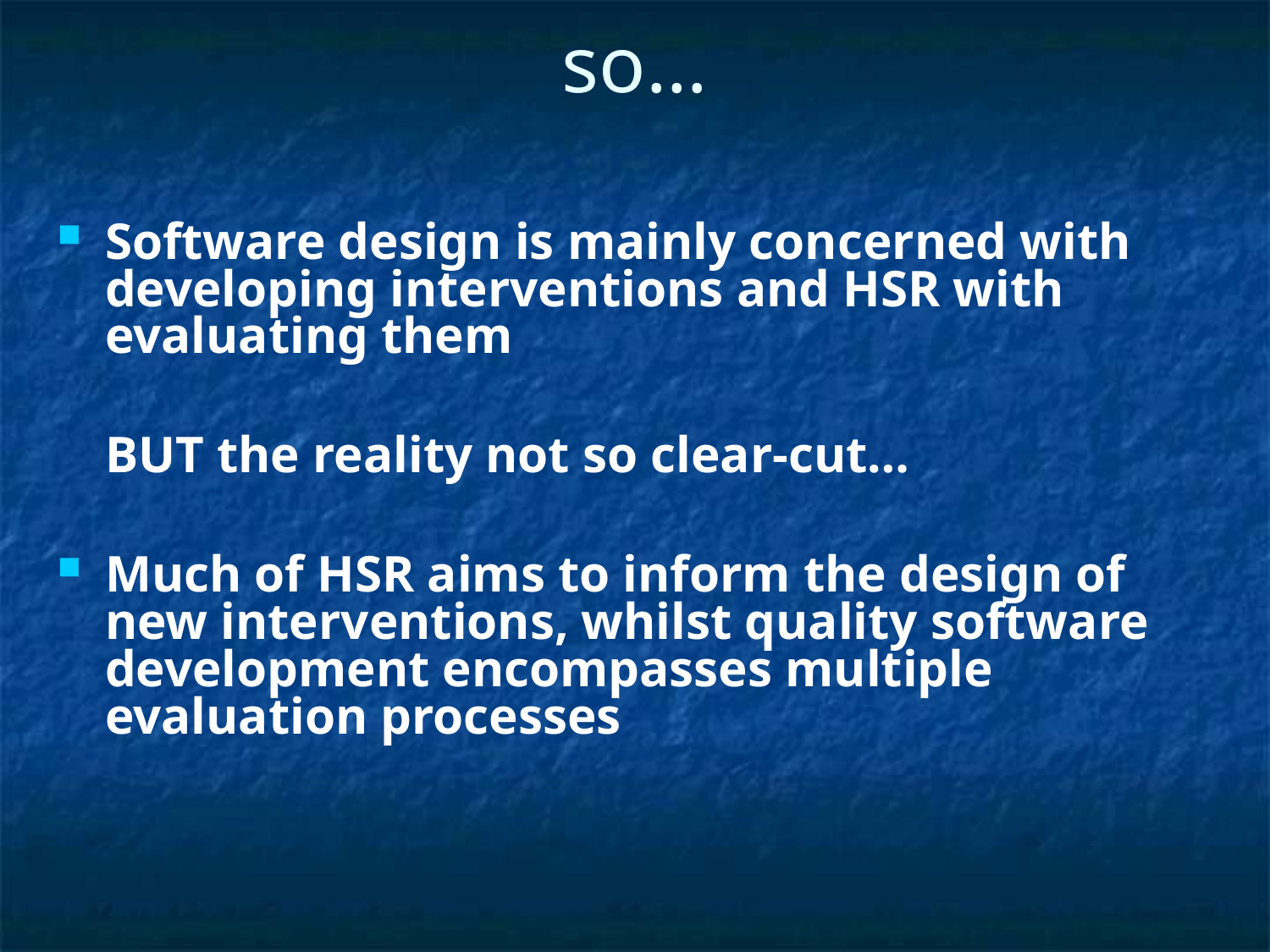

# so…
Software design is mainly concerned with developing interventions and HSR with evaluating them
BUT the reality not so clear-cut…
Much of HSR aims to inform the design of new interventions, whilst quality software development encompasses multiple evaluation processes

## Slide 12
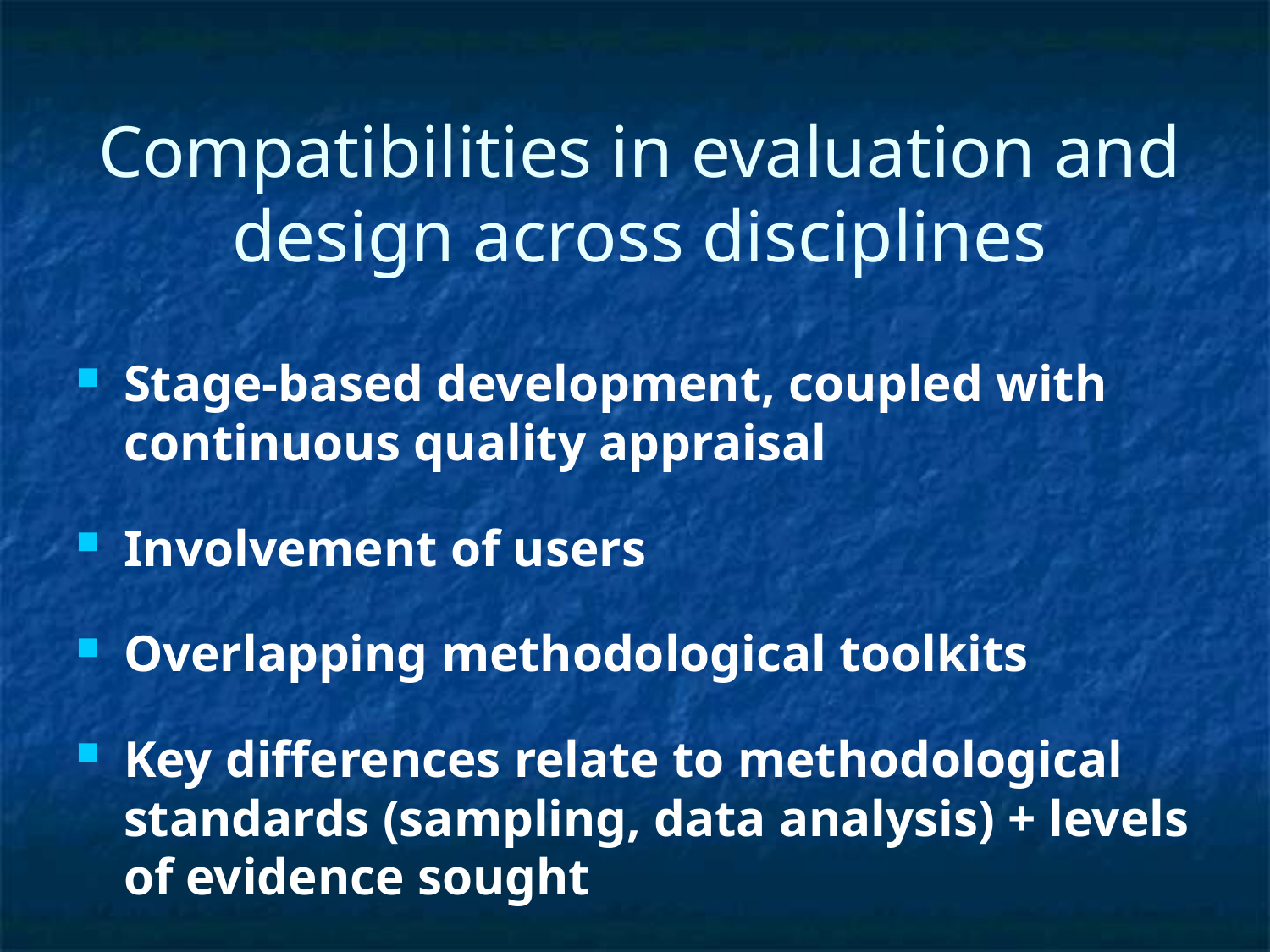

# Compatibilities in evaluation and design across disciplines
Stage-based development, coupled with continuous quality appraisal
Involvement of users
Overlapping methodological toolkits
Key differences relate to methodological standards (sampling, data analysis) + levels of evidence sought

## Slide 13
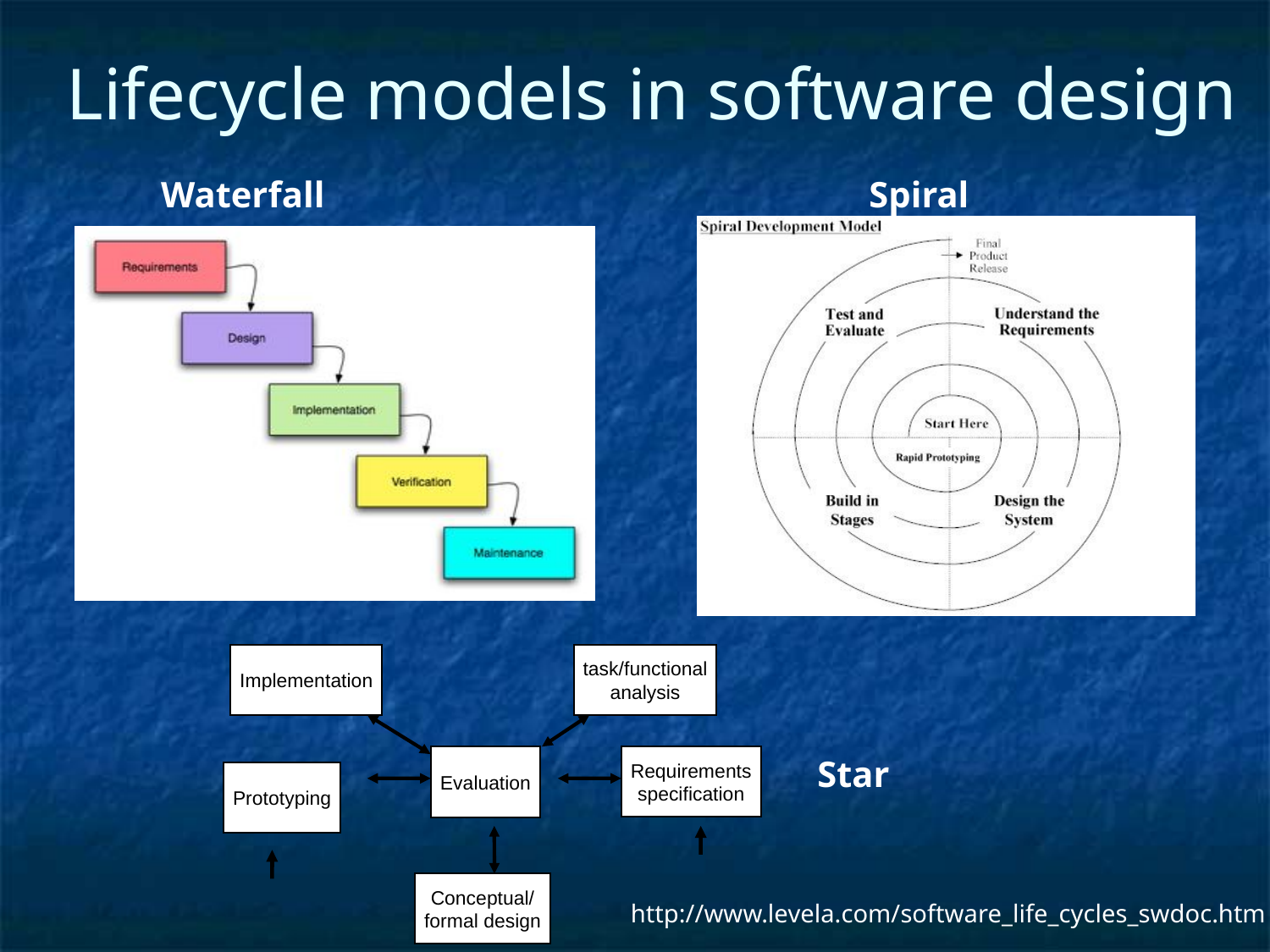

# Lifecycle models in software design
 Waterfall				 Spiral
Implementation
task/functional
analysis
Evaluation
Requirements
specification
Prototyping
Conceptual/
formal design
Star
 http://www.levela.com/software_life_cycles_swdoc.htm

## Slide 14
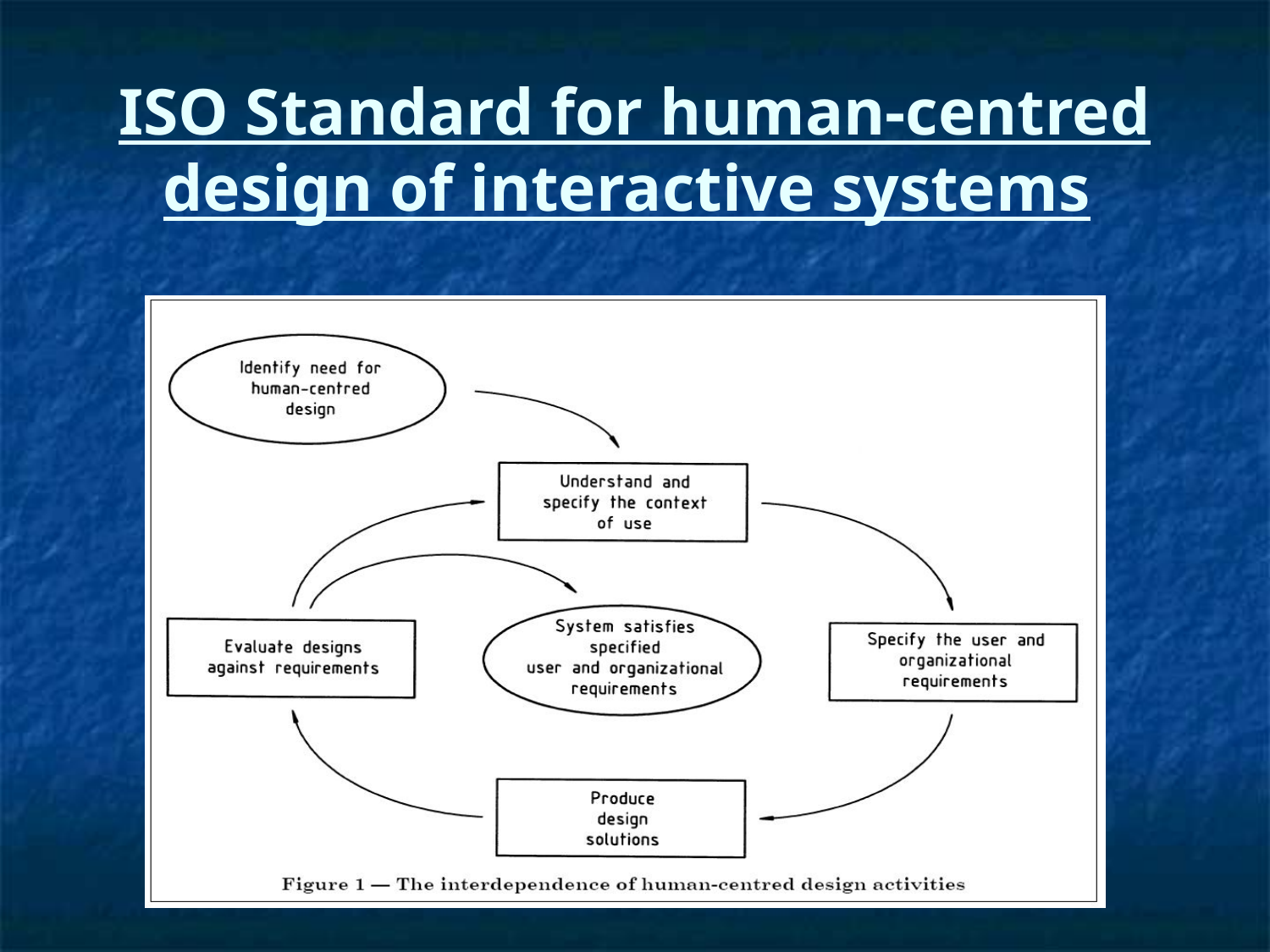

# ISO Standard for human-centred design of interactive systems

## Slide 15
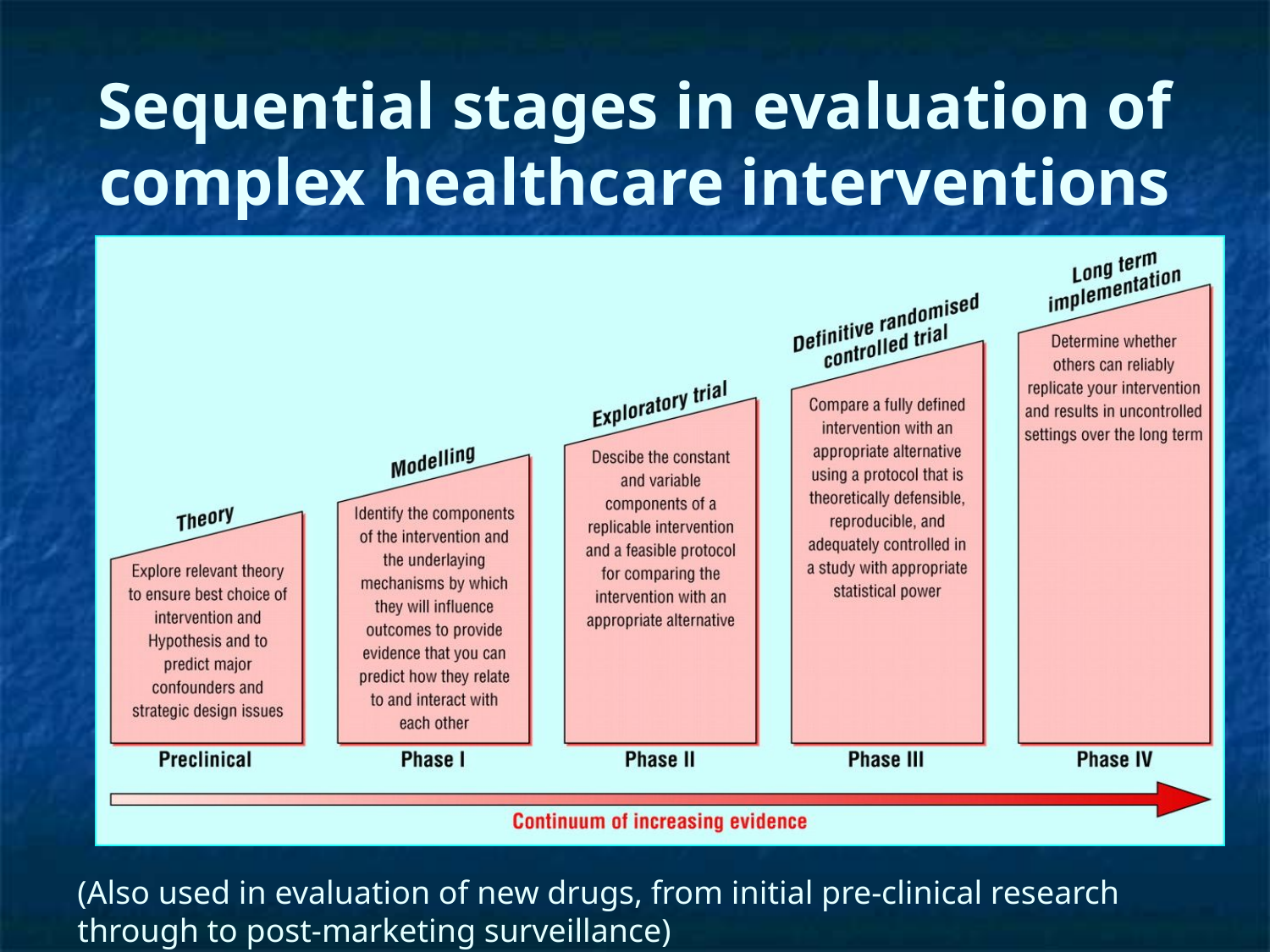

# Sequential stages in evaluation of complex healthcare interventions
(Also used in evaluation of new drugs, from initial pre-clinical research through to post-marketing surveillance)

## Slide 16
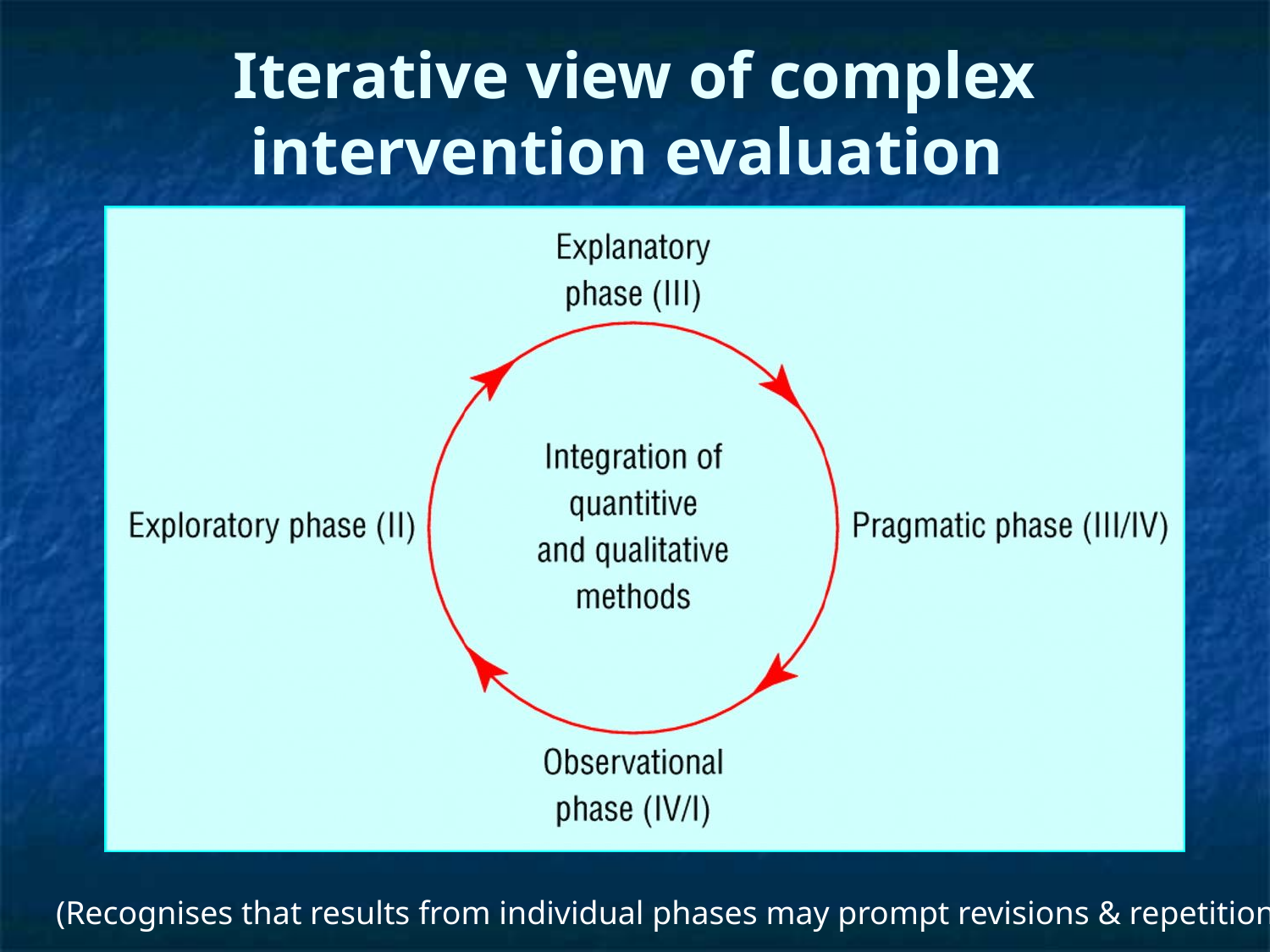

# Iterative view of complex intervention evaluation
(Recognises that results from individual phases may prompt revisions & repetition)

## Slide 17
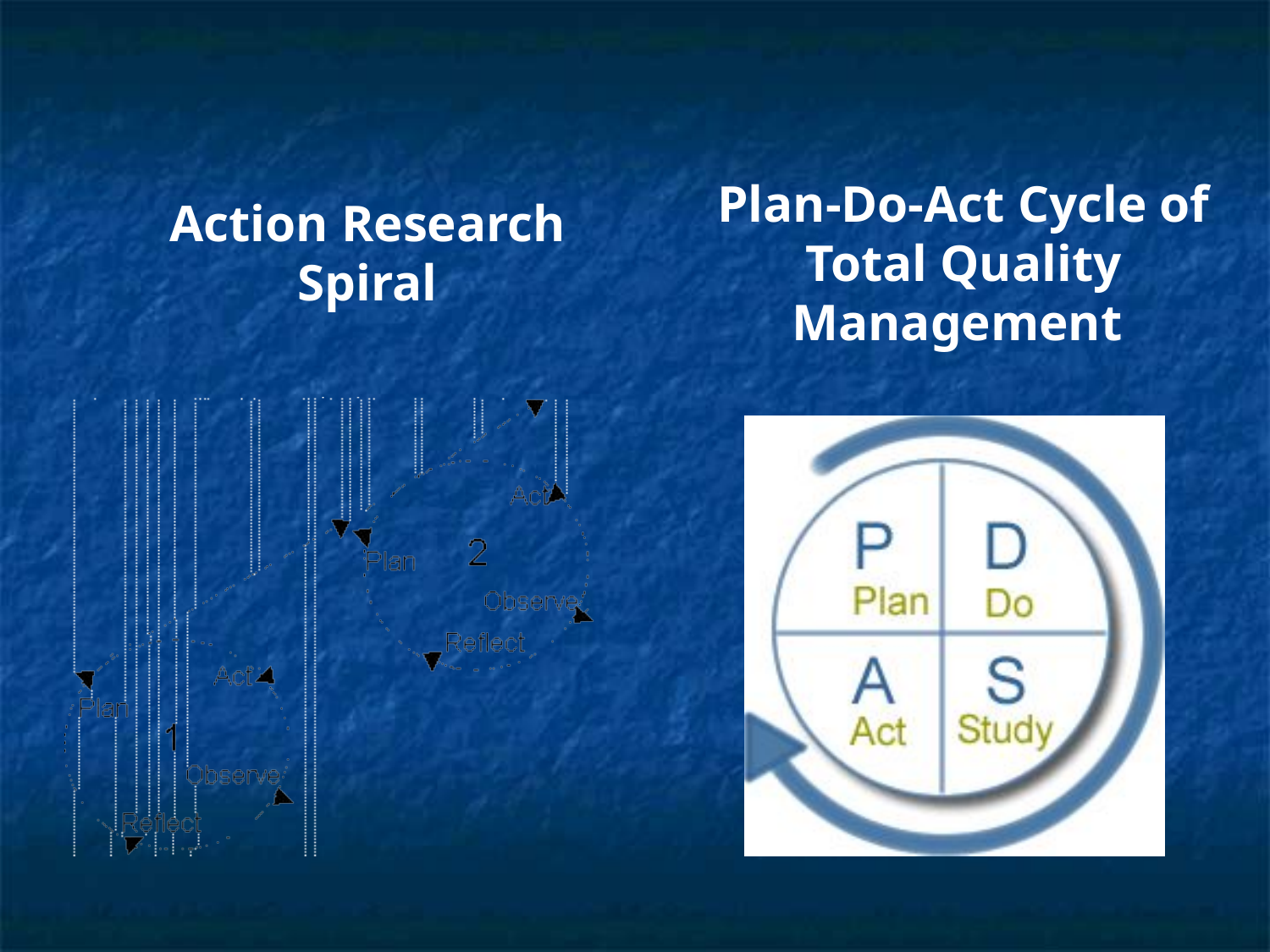

Plan-Do-Act Cycle of Total Quality Management
# Action Research Spiral

## Slide 18
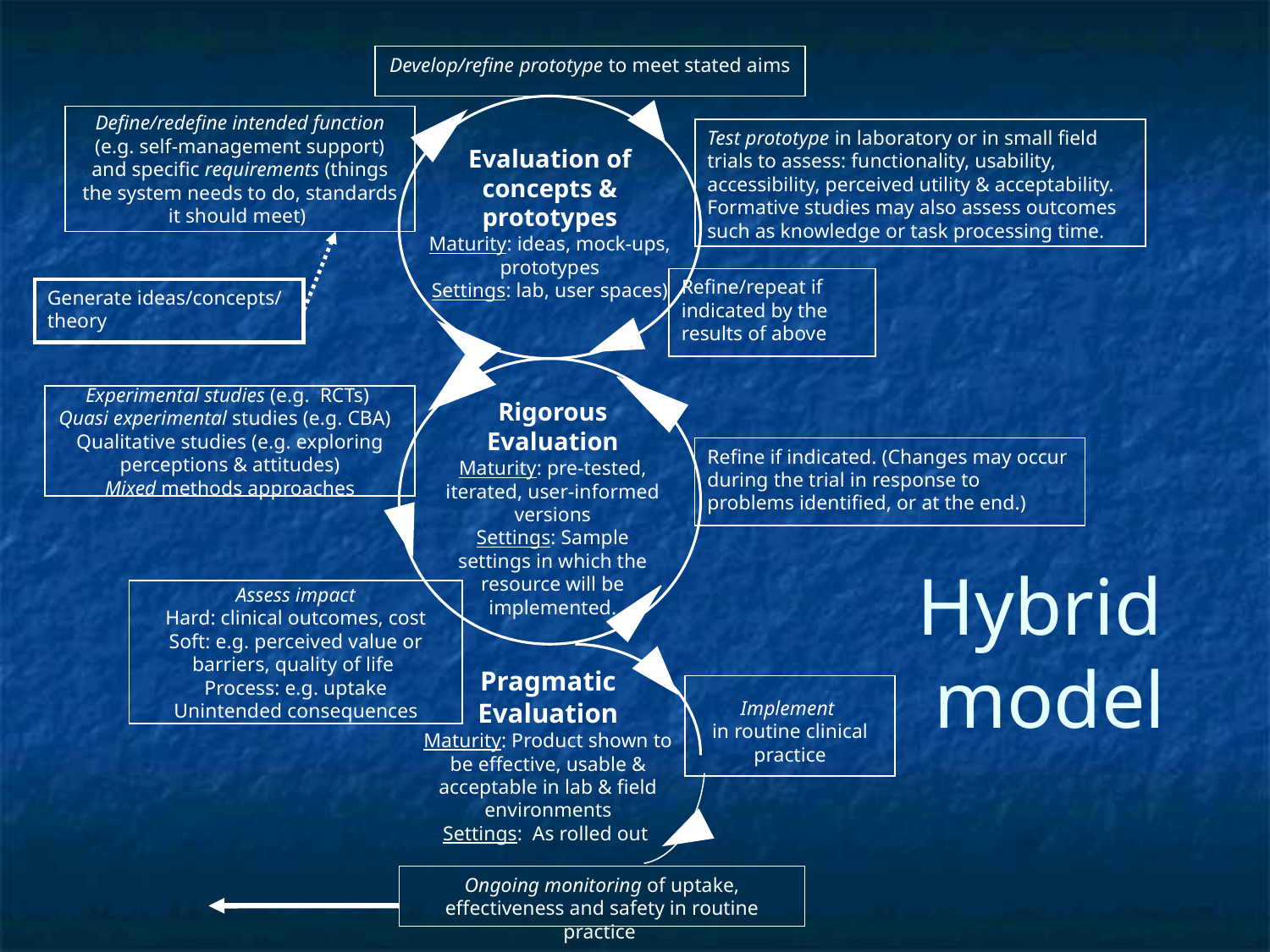

Develop/refine prototype to meet stated aims
Define/redefine intended function (e.g. self-management support) and specific requirements (things the system needs to do, standards it should meet)
Test prototype in laboratory or in small field trials to assess: functionality, usability, accessibility, perceived utility & acceptability. Formative studies may also assess outcomes such as knowledge or task processing time.
Evaluation of concepts & prototypes
Maturity: ideas, mock-ups, prototypes
Settings: lab, user spaces)
Refine/repeat if indicated by the results of above
Generate ideas/concepts/ theory
Experimental studies (e.g. RCTs)
Quasi experimental studies (e.g. CBA)
Qualitative studies (e.g. exploring perceptions & attitudes)
Mixed methods approaches
Rigorous
Evaluation
Maturity: pre-tested, iterated, user-informed versions
Settings: Sample settings in which the resource will be implemented.
Refine if indicated. (Changes may occur during the trial in response to problems identified, or at the end.)
# Hybrid model
Assess impact
 Hard: clinical outcomes, cost
Soft: e.g. perceived value or barriers, quality of life
Process: e.g. uptake
Unintended consequences
Pragmatic
Evaluation
Maturity: Product shown to be effective, usable & acceptable in lab & field environments
Settings: As rolled out
Implement
in routine clinical practice
Ongoing monitoring of uptake, effectiveness and safety in routine practice

## Slide 19
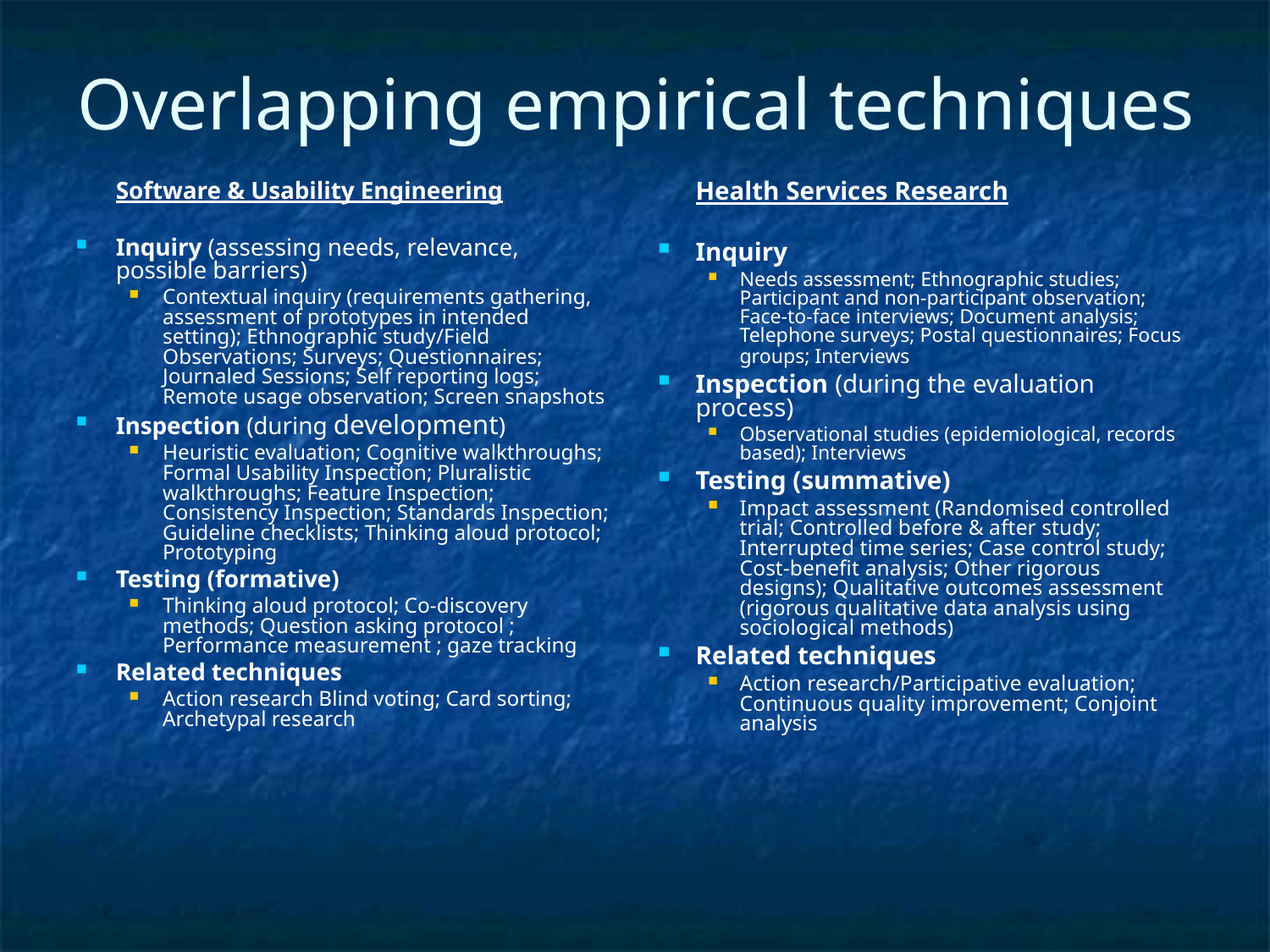

# Overlapping empirical techniques
Software & Usability Engineering
Inquiry (assessing needs, relevance, possible barriers)
Contextual inquiry (requirements gathering, assessment of prototypes in intended setting); Ethnographic study/Field Observations; Surveys; Questionnaires; Journaled Sessions; Self reporting logs; Remote usage observation; Screen snapshots
Inspection (during development)
Heuristic evaluation; Cognitive walkthroughs; Formal Usability Inspection; Pluralistic walkthroughs; Feature Inspection; Consistency Inspection; Standards Inspection; Guideline checklists; Thinking aloud protocol; Prototyping
Testing (formative)
Thinking aloud protocol; Co-discovery methods; Question asking protocol ; Performance measurement ; gaze tracking
Related techniques
Action research Blind voting; Card sorting; Archetypal research
Health Services Research
Inquiry
Needs assessment; Ethnographic studies; Participant and non-participant observation; Face-to-face interviews; Document analysis; Telephone surveys; Postal questionnaires; Focus groups; Interviews
Inspection (during the evaluation process)
Observational studies (epidemiological, records based); Interviews
Testing (summative)
Impact assessment (Randomised controlled trial; Controlled before & after study; Interrupted time series; Case control study; Cost-benefit analysis; Other rigorous designs); Qualitative outcomes assessment (rigorous qualitative data analysis using sociological methods)
Related techniques
Action research/Participative evaluation; Continuous quality improvement; Conjoint analysis

## Slide 20
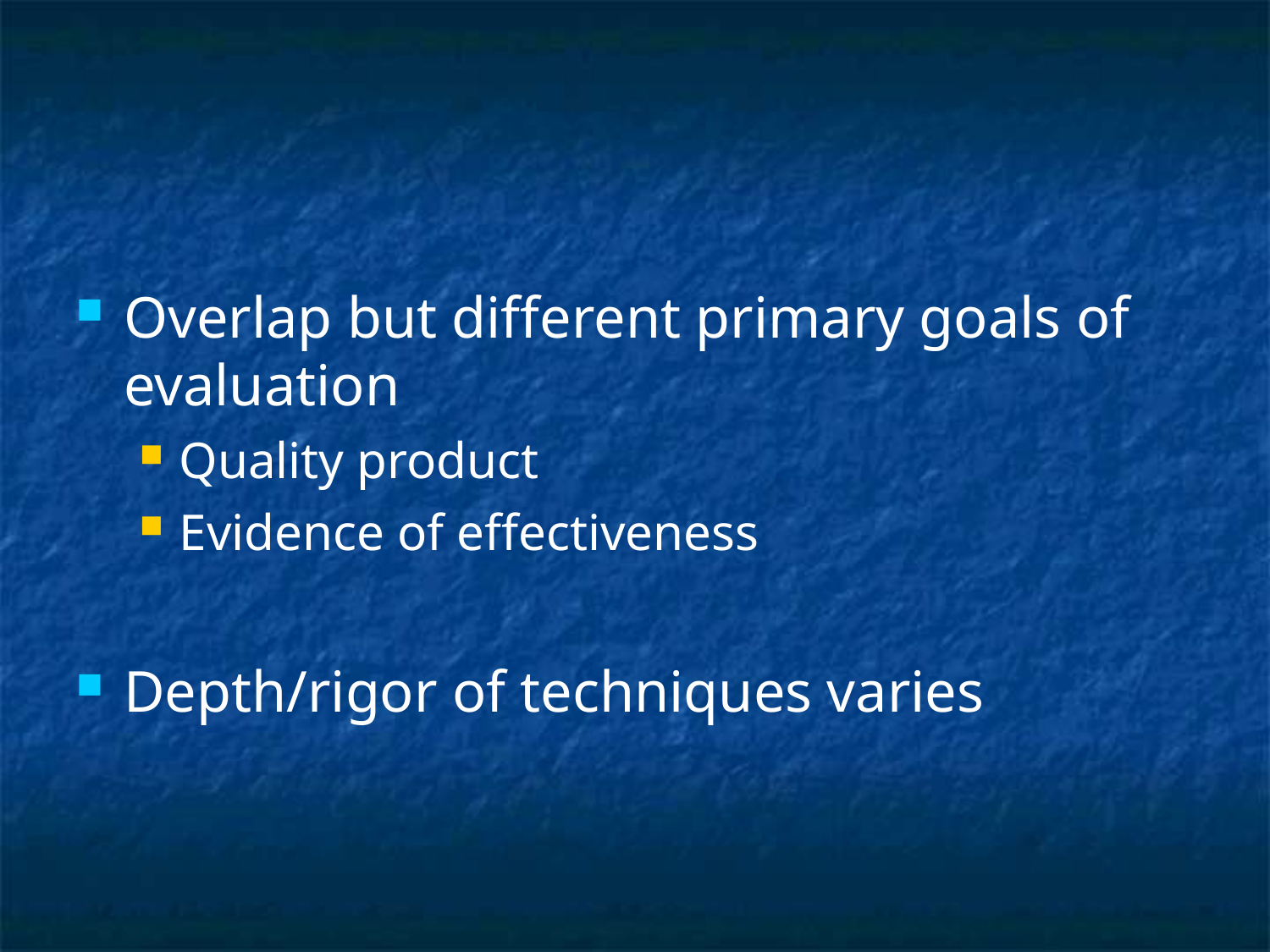

# Overlap but different primary goals of evaluation
Quality product
Evidence of effectiveness
Depth/rigor of techniques varies

## Slide 21
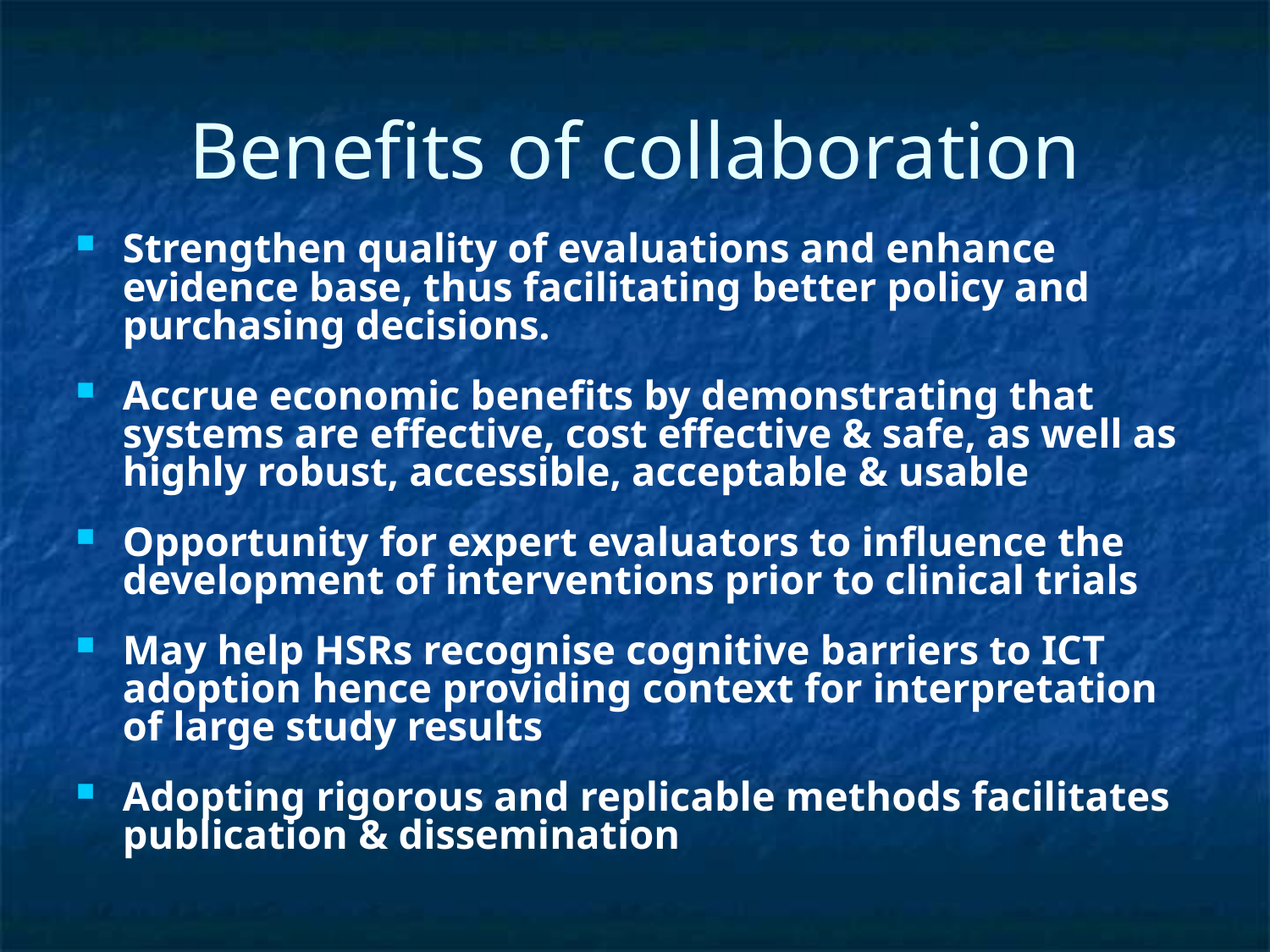

# Benefits of collaboration
Strengthen quality of evaluations and enhance evidence base, thus facilitating better policy and purchasing decisions.
Accrue economic benefits by demonstrating that systems are effective, cost effective & safe, as well as highly robust, accessible, acceptable & usable
Opportunity for expert evaluators to influence the development of interventions prior to clinical trials
May help HSRs recognise cognitive barriers to ICT adoption hence providing context for interpretation of large study results
Adopting rigorous and replicable methods facilitates publication & dissemination

## Slide 22
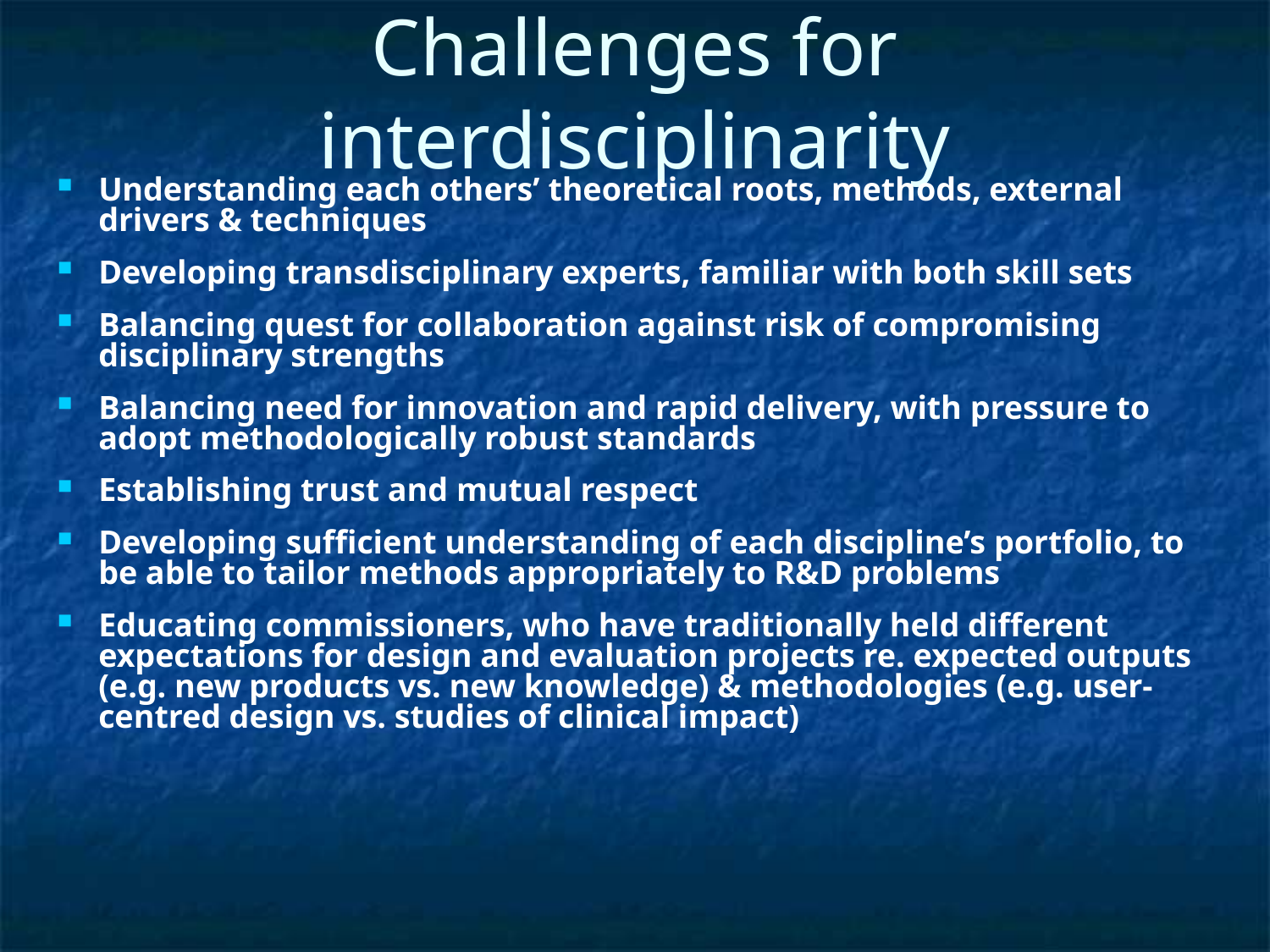

# Challenges for interdisciplinarity
Understanding each others’ theoretical roots, methods, external drivers & techniques
Developing transdisciplinary experts, familiar with both skill sets
Balancing quest for collaboration against risk of compromising disciplinary strengths
Balancing need for innovation and rapid delivery, with pressure to adopt methodologically robust standards
Establishing trust and mutual respect
Developing sufficient understanding of each discipline’s portfolio, to be able to tailor methods appropriately to R&D problems
Educating commissioners, who have traditionally held different expectations for design and evaluation projects re. expected outputs (e.g. new products vs. new knowledge) & methodologies (e.g. user-centred design vs. studies of clinical impact)

## Slide 23
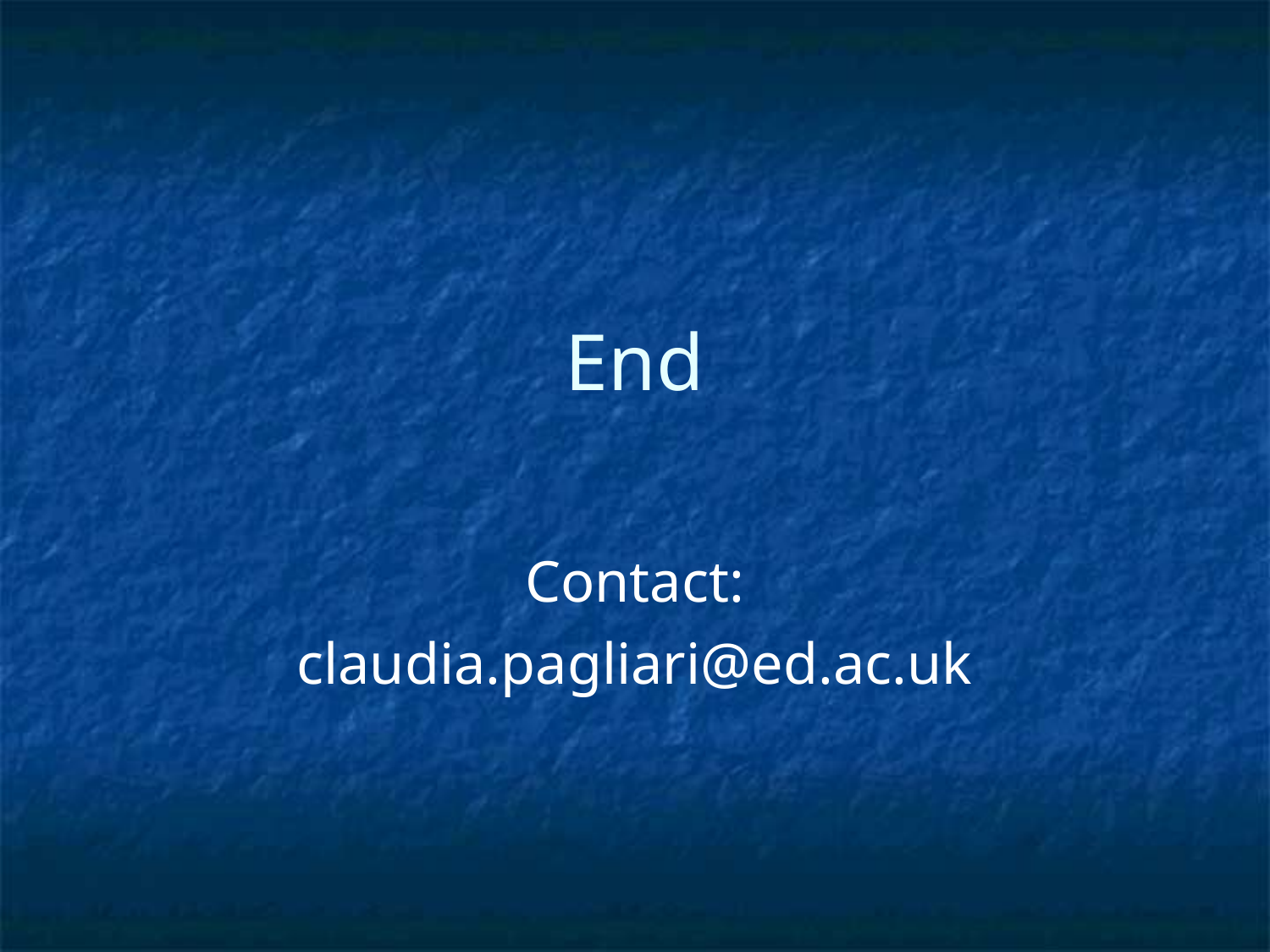

# End
Contact:
claudia.pagliari@ed.ac.uk
